# Eco-Genetic Structure of *Bacillus cereus sensu lato* Populations from Different Environments in Northeastern Poland

**DOI:** 10.1371/journal.pone.0080175

**Published:** 2013-12-02

**Authors:** Justyna M. Drewnowska, Izabela Swiecicka

**Affiliations:** Department of Microbiology, University of Bialystok, Bialystok, Poland; University of Exeter Medical School, United Kingdom

## Abstract

The *Bacillus cereus* group, which includes entomopathogens and etiologic agents of foodborne illness or anthrax, persists in various environments. The basis of their ecological diversification remains largely undescribed. Here we present the genetic structure and phylogeny of 273 soil *B. cereus s.l.* isolates from diverse habitats in northeastern Poland, with samplings acquired from the last European natural forest (Białowieża National Park), the largest marshes in Europe (Biebrza National Park), and a farm. In multi-locus sequence typing (MLST), despite negative selection in seven housekeeping loci, the isolates exhibited high genetic diversity (325 alleles), mostly resulting from mutation events, and represented 148 sequencing types (131 STs new and 17 STs already described) grouped into 19 complexes corresponding with bacterial clones, and 80 singletons. Phylogenetic analyses showed that 74% of the isolates clustered with *B. cereus s.l.* environmental references (clade III), while only 11 and 15%, respectively, grouped with isolates of clinical origin (clade I), and *B. cereus* ATCC 14579 and reference *B. thuringiensis* (clade II). Predominantly within clade III, we found lineages adapted to low temperature (thermal ecotypes), while putative toxigenic isolates (*cytK*-positive) were scattered in all clades of the marsh and farm samplings. The occurrence of 92% of STs in bacilli originating from one habitat, and the description of new STs for 78% of the isolates, strongly indicate the existence of specific genotypes within the natural *B. cereus s.l.* populations. In contrast to the human-associated *B. cereus s.l.* that exhibit a significant level of similarity, the environmental isolates appear more complex. Thus we propose dividing *B. cereus s.l.* into two groups, the first including environmental isolates, and the second covering those that are of clinical relevance.

## Introduction

*Bacillus cereus sensu lato* (*s.l*.) are Gram-positive endospore-forming bacilli that persist in different ecological environments [1], [2]. This group of bacteria includes various strains of *Bacillus thuringiensis*, an enthomopathogen successfully exploited in biocontrol worldwide [3], *Bacillus cereus sensu stricto (B. cereus s.s.)* which is associated with foodborne illness [4], and *Bacillus anthracis*, the etiologic agent of anthrax [5]. *Bacillus cereus s.l.* also include psychrotolerant *Bacillus weihenstephanensis* [6], thermotolerant *Bacillus cytotoxicus* [7], as well as *Bacillus mycoides* and *Bacillus pseudomycoides* that form characteristic rhizoidal colonies on solid media [8]. The species-specific properties of these bacteria are generally based on plasmid-borne signatures [5], entomopathogenicity [9], and the ability to synthesize cereulide [10]. In contrast to extra-chromosomal profiles, the chromosomes of these bacilli exhibit a high level of synteny [11], thus giving rise to controversy regarding their taxonomy. Based on genetic evidence, *B. thuringiensis*, *B. cereus s.s.* and *B. anthracis* have been designated as one species [12] or are contained in one genetic cluster [13], although others have reported that at least *B. anthracis* represents an independent taxon [14]. From an ecologic perspective, the taxonomy of *B. cereus s.l.* is even more complicated when variations in their symbiotic associations, including species-specific virulence, are considered [1], [5], [15], [16]. For example, cereulide, typically associated with the emetic strains of *B. cereus s.s.*, is also produced by *B. weihenstephanensis* [17], while *B. cereus s.s.* may cause clinical symptoms similar to those of inhalation anthrax [18]. Other properties attributed to a particular species may not be intrinsically unique, for example, psychrotolerance, a primary feature of *B. weihenstephanensis* [6], has recently been reported among *B. thuringiensis* isolates [19], [20].

Data reported over the last decade have revealed the importance of the environment to bacterial adaptation, diversification, and evolution [21], [22], [23], [24], [25]. Soil, which is the primary niche for *B. cereus s.l.* [2], [26], creates favorable conditions for these processes to occur due to its heterogeneity in nutrients, particle size, pH, humidity and microbiota [22], [27], [28]. Thus, so called ecotypes, defined as a cohesive group of bacteria that are ecologically similar to one another and are closely related at the molecular level, evolve and proliferate as a result of acquired genetic determinants or favorable mutations that confer selective advantages conducive to a particular niche or ecosystem [29]. From this point of view, ecotypes represent distinct evolutionary lineages within a particular species [21].

The mechanisms involved in ecological diversification of *B. cereus s.l.* are poorly understood. Considering the group's biological and ecologic properties we postulate that (1) in particular habitats distinct genotypes (ecotypes) of *B. cereus s.l.* occur, and (2) environmental *B. cereus s.l.* are highly related genetically and should be classified as one species. To test these hypotheses, we assessed the genetic structure and phylogenetic relationship of soil isolates of *B. cereus s.l.* using the multi-locus sequence typing (MLST) approach based on seven housekeeping genes. These isolates were collected from geographically and ecologically distinct locations in northeastern Poland, namely, (i) Białowieża National Park (Białowieża NP), the last European natural forest with a primeval character and limited human activity (a World Heritage site and a biosphere reserve), (ii) Biebrza National Park (Biebrza NP), the largest of Europe's marshes and also with limited human activity, located in the Biebrza River basin, and (iii) the agricultural soil in Jasienowka, a small village south of Podlasie province. The results of our study provide new insights into population structure of environmental *B. cereus s.l.* and address key issues regarding their ecology and phylogeny.

## Materials and Methods

### Soil sampling, and chemical and physical analysis of soil

**Figure 1:**
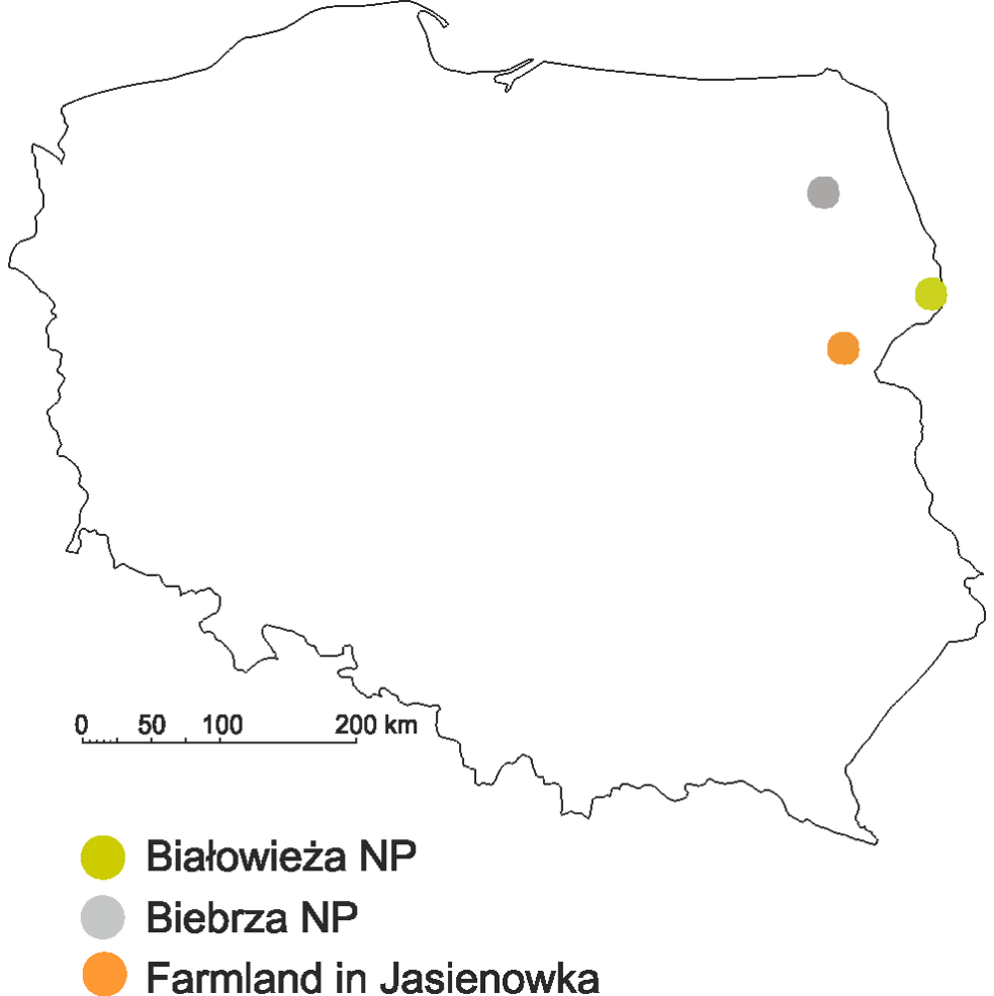
Geographic map of Poland with locations of soil sampling. Soil samples were collected in Białowieża National Park (Białowieża NP; N 52°42′, E 23°54′), the natural forest with no human activity, in marshes of Biebrza National Park (Biebrza NP; N 53°36′, E 22°56′) with limited human activity, and a farmland in Jasienowka (N 52°30′, E 22°58′) in northeastern Poland.

Soil samples were obtained from Białowieża NP (forest bacilli), Biebrza NP (marsh bacilli), and agricultural land in Jasienowka (farm bacilli), northeastern Poland. The marsh collections consisted of soil samples taken adjacent to water. All samples from the parks were collected with consent according to the Nature Conservation Act adopted on 16 April, 2004 by Polish Parliament (Parliament Diary 2004, No. 92: 880). The owner of the farm also permitted collection of soil samples for our study. The locations of the sampling areas are ∼60–200 km apart (Figure 1). At each location 60 to 90 soil samples were collected from sites distributed over an area of about 50,000 m^2^. Samples were analyzed by the Institute of Soil Science and Plant Cultivation in Puławy, Poland, for pH, organic content, humic substances, carbon, nitrogen, phosphorus, calcium, and sulfur.

### Isolation and identification of *B. cereus sensu lato*

*B. cereus s.l.* were isolated as described previously [30]. Bacilli showing rhizoidal growth on the MYP agar (Oxoid, Basingstoke, UK), a selective medium for the *B. cereus* group, were classified as *B. mycoides/B. pseudomycoides* (referred hereafter as *B. mycoides*). Bacteria forming rough and dry colonies with a violet-pink background surrounded by egg yolk precipitation, were designated as *B. cereus* or *B. thuringiensis* if parasporal crystals were observed under phase-contrast microscopy (Olympus BX61). From each sample, only one isolate of *B. mycoides* and *B. cereus s.s.* or *B. thuringiensis* were selected for further study. Occasionally, two isolates of the same species or those classified as *B. cereus s.s.* or *B. thuringiensis*, but showing visible differences in colony appearance on MYP, were selected from one sample for further analysis. Each isolate was subsequently screened for hemolytic activity on Columbia Blood Agar (Oxoid) at 30°C and for psychrotolerance and thermotolerance by growth in Luria-Bertani (LB) broth at 7°C and 50°C, respectively. The diagnostic features used for species-level classification of the *B. cereus s.l.* isolates are provided in Table S1. Based on colony morphology and microscopic observation, ∼30 isolates of *B. cereus s.s.*, *B. thuringiensis*, and *B. mycoides* from each location were selected randomly for further analysis.

### DNA extraction

Genomic DNA was extracted from overnight LB broth culture using the DNeasy Blood and Tissue Kit (Qiagen GmbH, Hilden, Germany) and the QIAcube automat (Qiagen). The quantity and purity of DNA were determined using the NanoDrop 2000 Spectrophotometer (Thermo Fisher Scientific, Wilmington, USA).

### Detection of the δ-endotoxins genes and the *cytK* gene

All isolates were screened for the presence of *cytK* encoding cytotoxin K in *B. cereus s.l.* [16] and the most frequently occurring genes encoding δ-endotoxins in *B. thuringiensis*: *cry1*, *cry2*, *cry4*, and *cry9* [31], as described by Ben-Dov et al. [32], [33] (PCR primer sequences are provided in the Table S2). PCR (15 µl reactions containing 0.35 U *Taq* DNA polymerase (MBI Fermentas), 150 ng of DNA, 0.5 mM of each dNTP, 1.5 mM MgCl_2_, and 0.5 µM of each of the primers] was performed using the Veriti 96-Well thermal cycler (Applied Biosystems, Foster City, USA), and amplicons were analyzed in the capillary electrophoresis system QIAxcel (Qiagen). *B. thuringiensis* subsp. *kurstaki* HD1, *B. thuringiensis* subsp. *aizawai* HD133, and *B. thuringiensis* subsp. *israelensis* HD567 (BGSC, *Bacillus* Genetic Stock Centre, Ohio State University, Ohio, USA) were used as reference strains for the *cry* genes. *B. cereus* ATCC 14579 (ATCC, American Type Culture Collection) was used as the reference strain for *cytK*.

### Real-Time PCR of *cytK*

RNA was isolated using the Total RNA Mini Plus Kit (A&A Biotechnology, Gdynia, Poland), and cDNA was prepared with the High Capacity cDNA Reverse Transcription Kit (Applied Biosystems) according to the manufacturer's instructions. Reactions were performed in the Veriti 96 Well thermal cycler (Applied Biosystems) in a final volume of 20 µl, as follows: 10 min at 25°C, 120 min at 37°C, 5 min at 85°C, and 5 min at 4°C. The cDNA was analyzed using the Step One Plus Real-Time PCR System (Applied Biosystems). The reactions were performed using the Real-Time 2×PCR Master Mix SYBR A Kit (A&A Biotechnology) in 20 µl of the reaction mixture, containing 10 µl of RT 2×PCR Master Mix, 0.1–1 µM of each forward and reverse *cytK* primers (Table S2), 10 pg – 1 µg template cDNA and water added to a final volume of 20 µl. The thermal protocols for *cytK* and *udp* (uridine phosphorylase), genes used as a reference [34], were as follows: 95°C for 3 min (*cytK*) and 2 min (*udp*); 40 cycles of 95°C for 15 sec (*cytK*) and 30 sec (*udp*), 60°C (*cytK*) and 57°C (*udp*) for 30 sec, 72°C for 45 sec (*cytK*) and 1 min (*udp*). Threshold cycle (*C_T_*) was normalized to the *C_T_* of the *udp* gene amplified from the corresponding sample. The expression of *cytK* was calculated using the Pfaffl's method [35] with comparison to the reference *B. cereus* ATCC 14579.

### Multi-locus sequence typing (MLST)

All isolates were characterized by the MLST scheme using seven housekeeping loci, *glpF* (glycerol uptake facilitator protein), *gmk* (putative guanylate kinase), *ilvD* (dihydroxy-acid dehydratase), *pta* (phosphate acetyltransferase), *pur* (phosphoribosylamino-imidazolecarboxamide), *pycA* (pyruvate carboxylase), and *tpi* (triosephosphate isomerase). The 534–599 bp fragments originating from these loci were amplified as described previously [30] in the Veriti 96-Well thermal cycler (Applied Biosystems) with the pairs of primers recommended in the *B. cereus* PubMLST database (http://pubmlst.org/bcereus) (see Table S2). Amplicons were analyzed by the capillary electrophoresis QIAxcel system (Qiagen). The products were purified with the CleanUp Kit (A&A Biotechnology, Gdynia, Poland) and sequences were determined with the ABI3500 automated sequencer (Applied Biosystems) using Big Dye Terminator cycle sequencing Kit (Applied Biosystems).

The MLST scheme sequences (348–504 bp) were assembled with the BioEdit Sequence Alignment Editor version 7.0.1 software. Each unique sequence was assigned an arbitrary allele number by reference to the *B. cereus* group MLST database. The combination of allele numbers for all seven loci of a given isolate allowed assessing the specific sequence type (ST), regarded also as genotype. New allele sequences and STs were submitted to the *B. cereus* PubMLST database. The χ^2^ test with Bonferroni correction, performed with the R version 2.15.2 software, was used for pairwise analysis of statistical differences in proportions of the new STs in the habitats. The null hypothesis of the test is that the proportions are even. The significance level was set at 0.05.

### Diversity of the loci

In order to assess loci diversity and their significance in selection, allele frequencies of each loci and the number of polymorphic sites were calculated using the DnaSP version 5 software [36]. The same software was also used to calculate pairwise ratios of nonsynonymous (*dN*) substitutions to synonymous (*dS*) substitutions (*dN/dS*) according to Nei and Gojobori [37]. This statistic allows measuring the significance of selection, as follows: *dN/dS*<1, purifying selection; *dN/dS* = 1, neutral selection; *dN/dS*>1, positive selection [38]. The relative impact of homologous recombination on the variation of the *B. cereus s.l.* populations was estimated by calculation of the r/m ratio using ClonalFrame software version 1.2 [39]. The analysis was performed on the complete dataset (N = 273) and with regard to each environment with four independent runs. The basis of 100,000 iterations including 50,000 burn in iterations was used in all runs of the algorithm.

### Population genetic analysis

The sequence types (STs) were assigned to clonal complexes using PHYLOViZ v1.0 [40] with goeBURST algorithm and 1,000 bootstrap resampling, according to Feil et al. [41]. Clonal complexes (CCs) were defined as Single Locus Variants (SLV) of two or more independent isolates that shared identical alleles at six or seven loci. To assess the correlation between alleles in the populations and the clonal versus panmixia status [42], the standard index of association (I^S^_A_) was calculated using the START version 2 software [43]. This statistic allowed for estimating the degree of linkage disequilibrium among loci. I^S^_A_∼0 indicates equilibrium between recombination and mutation rates. I^S^_A_>1 means low rate of recombination in relation to mutation. The χ^2^ test with Bonferroni correction, performed with the R version 2.15.2 software, was used for pairwise analysis of the statistical differences in proportions of bacilli forming clonal complexes with regard to their species designation. The null hypothesis of the test is that the proportions are even. The significance level was set at 0.05.

### Phylogenetic analysis

Phylogenetic trees based on the concatenated loci were constructed for each population and for all isolates jointly with the MEGA4 software using the Neighbor-Joining method (NJ). Branch quality was evaluated using 1,000 replicates bootstrap test [44]. Altogether 28 reference sequences from the *B. cereus* MLST database were used for comparative analysis.

## Results

### Occurrence of *B. cereus s.l.* in different types of soil

The estimated density of *B. cereus s.l.* isolates from the farm, and Białowieża and Biebrza national parks (NPs) were, respectively, 0.7±0.7×10^5^, 1.4±1.7×10^5^ and 1.5±2.2×10^5^ (Table 1). At each location, the density differed significantly among samplings. Moreover, while *B. cereus s.s.* and *B. thuringiensis* were isolated from each sample, *B. mycoides* was present in all marsh and farm samples, but only in 77% of forest samples. Chemical analysis revealed significant differences in nutrients from different locations, for example, the amount of humic substances and organic matter were ∼20 times higher in soil from the NPs than the farm soil, which allowed the classification of the soil in the parks as organic, and the farm as mineral (Table 1).

**Table 1:** Soil types, chemical properties and density of *B. cereus sensu lato* in the samples.

	Soil		Content in air-dry soil [%]							Number of
Origin	type^a^	pH	SOM^b^	HS^c^	C	N	P org.	Ca	S	*B. cereus s.l.* d
Białowieża	O	4,75	24,1	21,4	14,8	0,753	0,041	0,39	0,06	1.4±1.7×10^5^
National Park										
Beira	O	7,15	17,3	18,9	13,1	1,005	0,126	1,87	0,24	1.5±2.2×10^5^
National Park										
Farmland in	M	5,25	1,68	0,98	0,66	0,062	0,037	0,127	0,01	0.7±0.7×10^5^
Jasienowka										

a M, mineral soil; O, organic soil;

b soil organic matter;

c humic substances;

d given as an average CFU per gram of soil.

### Genetic diversity of the environmentally different *B. cereus s.l.* populations

**Figure 2:**
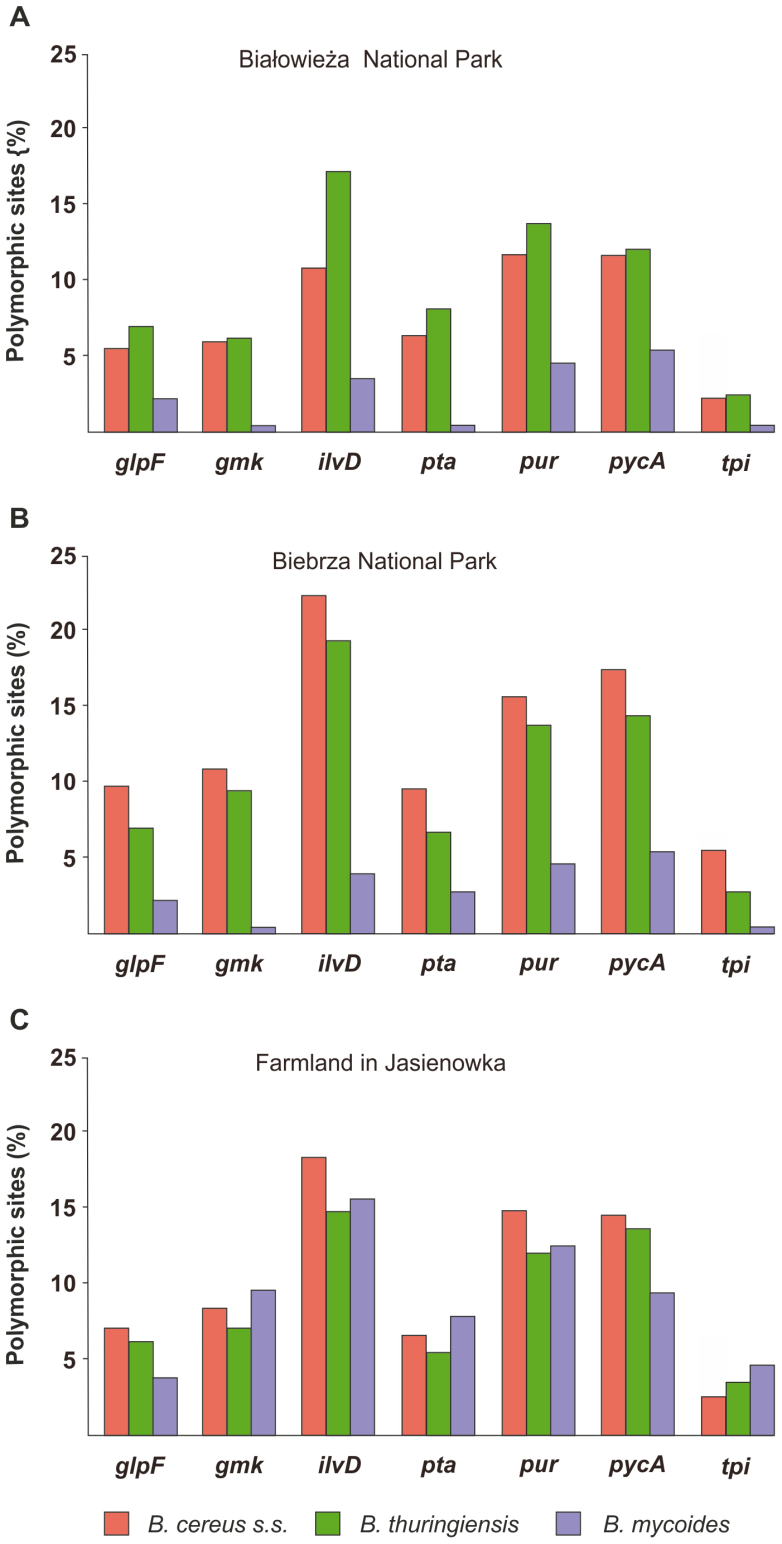
Genetic diversity in housekeeping genes within *B. cereus s.l.* originated from three locations in northeastern Poland. The percentage of the polymorphic sites was much higher in *B. cereus* and *B. thuringiensis* than in *B. mycoides* originating from Białowieża National Park (A) and Biebrza National Park (B). Contradictory for bacteria isolated from the farm soil (C), the percentage of the polymorphic sites was similar among bacilli classified to the three species.

The 273 *B. cereus s.l.* isolates were analyzed with the MLST approach to assess their genetic relationship and ecological diversification. The characteristic of each locus is presented in Table 2 and Figure 2 (additional data are shown in Table S3). The polymorphic sites ranged from 307 to 392 among the forest and marsh isolates, and was much higher in *B. cereus s.s.* and *B. thuringiensis* than *B. mycoides* originating from the parks, that was similar for all three species isolated from the farm samplings. However, the number of polymorphic sites among the farm *B. mycoides* was influenced by the number of polymorphic sites in two strains further classified to a separate phylogenetic clade (see below). Altogether 325 alleles were identified. The ratio of nonsynonymous to synonymous mutations (*dN/dS*) was less than one for all loci in all isolates, indicating purifying selection among the genes. The relative frequency of recombination and mutation among all *B. cereus s.l.* isolates (N = 273; 95% credibility interval) varied from 0.9 to 1.5. When single environments were considered the recombination to mutation ratio was slightly higher among strains from Białowieża NP (1.2–2.7) than those from Biebrza NP (0.8–1.9) and farmland (0.8–1.8).

**Table 2:** Genetic diversity in the seven loci within three *B. cereus sensu lato* populations from northeastern Poland.

Bia^3^owieża National Park	Biebrza National Park	Farmland
N = 93 (*B.c.*-25; *B.t.*-38; *B.m.*-30)^a^	N = 95 (*B.c.*-39; *B.t.*-26; *B.m.*-30)^a^	N = 85 (*B.c.*-38; *B.t.*-23; *B.m.*-24)^a^
ST = 57 (56)^b^	ST = 55 (43)^b^	ST = 49 (40)^b^

Locus	Polymorphic sites	Alleles^c^	Index *d*N/*d*S^d^	Polymorphic sites	Alleles^c^	Index *d*N/*d*S^d^	Polymorphic sites	Alleles^c^	Index *d*N/*d*S^d^
*glpF*	34	22 (10)	0.046	41	24 (8)	0.101	34	21 (3)	0.125
*gmk*	41	11 (7)	0.023	57	16 (4)	0.018	86	20 (7)	0.017
*ilvD*	73	20 (13)	0.014	94	25 (14)	0.015	103	17 (4)	0.017
*pta*	39	24 (12)	0.020	44	24 (8)	0.019	54	21 (5)	0.024
*pur*	49	21 (12)	0.031	62	24 (9)	0.018	80	24 (8)	0.016
*pycA*	56	14 (6)	0.030	67	18 (5)	0.029	80	21 (6)	0.030
*tpi*	15	12 (5)	0.082	27	21 (8)	0.076	32	17 (4)	0.070
Total	307	124 (65)	0.030	392	152 (56)	0.032	389	141 (37)	0.033

a *B.c.*, *Bacillus cereus s.s.*; *B.t.*, *Bacillus thuringiensis*; *B.m.*, *Bacillus mycoides*.

b ST, sequencing type; number of new STs described in this study is given in the parentheses.

c Number of new alleles described in this study are given in the parenthesis.

d Ratio of nonsynonymous (*d*_N_) to synonymous (*d*_S_) substitutions per nucleotide site where *d*N/*d*S<1 indicates that the loci is subjected to purifying selection.

The isolates were classified into 148 STs, 131 new STs (N = 212) and 17 STs (N = 61) already described in the MLST database. Almost all STs (56/57) detected in bacilli originating from Białowieża NP were found to be new, whereas with the marsh and farm isolates the number of new STs was 43/55 and 40/49, respectively. According to the χ^2^ test, the proportion of the new STs to all STs of bacilli from Białowieża NP (86/93) differed significantly from the corresponding proportions in samples from Biebrza NP (61/95) and Jasienowka farm (65/85), with p-value of 0.0076 and 0.0293, respectively. However, the proportions in the samples from the latter two habitats did not differ significantly (p-value = 1).

Altogether 105 STs were present only once, while the most numerous, ST222, ST625, and ST624, were detected 27, 19, and 13 times, respectively. With regard to the environment, 50, 47, and 40 STs were associated only with the forest, marsh, and farm isolates, respectively. Additionally, nine STs (N = 41) were found in two environment simultaneously: ST646 and ST665 in the forest and marsh isolates; ST624, ST650 and ST712 in bacteria isolated from the forest and the farm; ST196, ST218, ST410; and ST647 with the marsh and farm isolates. Only ST222 (N = 27) and ST625 (N = 19) contained bacteria from all three environments. Considering the species classification, 68, 54, and 19 STs were identified among *B. cereus s.s.*, *B. thuringiensis* and *B. mycoides*, respectively. *B. cereus* and *B. thuringiensis* originating from the same environment were found simultaneously in five STs (ST657, ST683, ST705, ST708, ST737), while *B. cereus* and *B. mycoides* were present in ST222 (two *B. cereus* plus 25 *B. mycoides*) and ST624 (one *B. cereus* and 12 *B. mycoides*). *B. thuringiensis* with *B. mycoides* or three species together did not occur in one ST.

### Isolate-based analysis of population genetic structure

**Figure 3:**
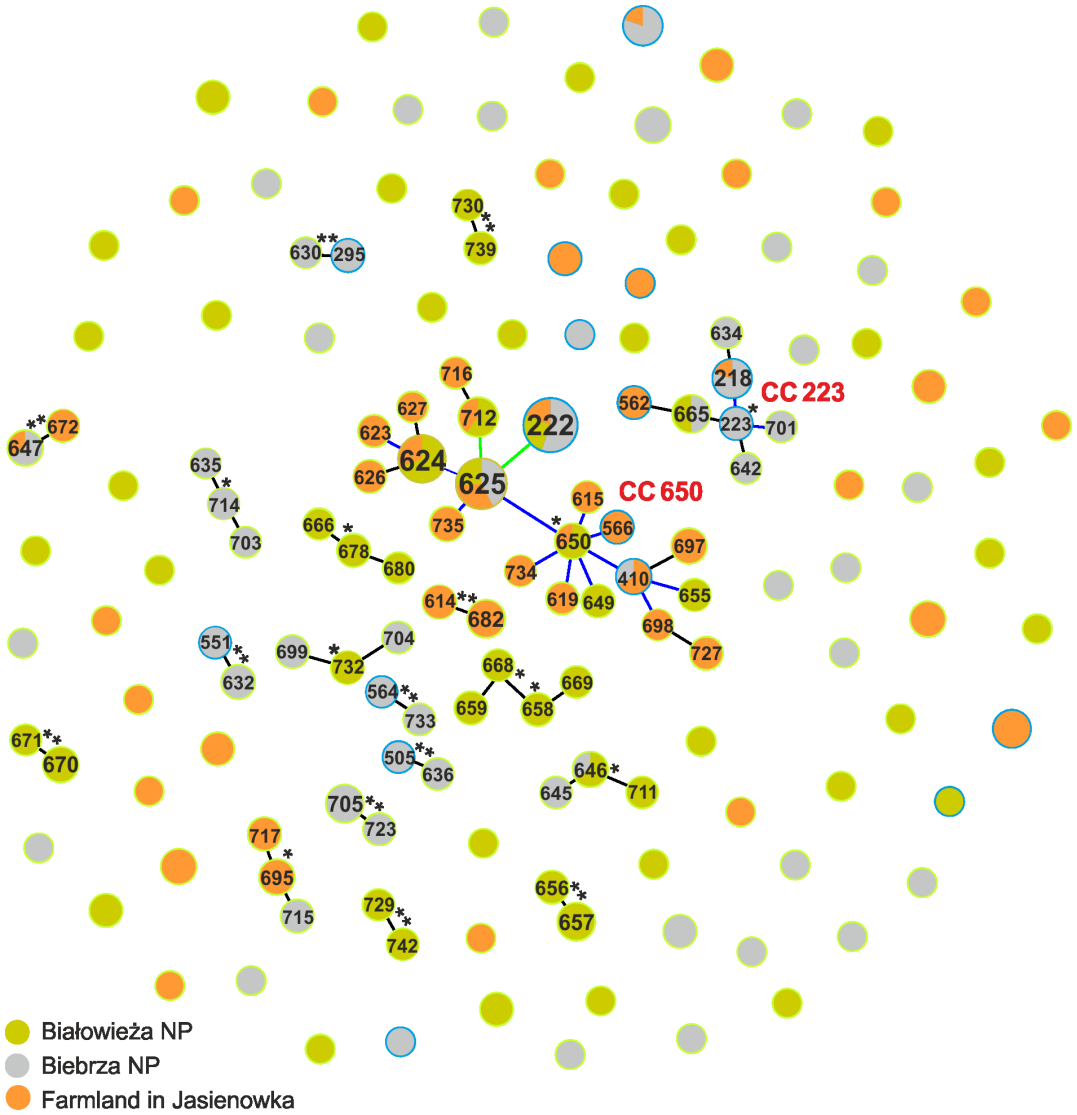
geoBURST analysis showing the clonal assignment of STs present in *B. cereus s.l.* Bacteria originated from Białowieża National Park, Biebrza National Park, and the Jasienowka farm in northeastern Poland. The CCs are named based on the ST assigned as a founder genotype (marked with a star) of the complex. The relative size of the circles indicates their prevalence among the *B. cereus s.l.* isolates. New STs characterized in this study are accentuated by a green halo, while STs present in the MLST database are accentuated by a blue halo.

The goeBURST approach allowed assigning the isolates to 19 clonal complexes (CCs) containing 68 STs (N = 172), and 80 singletons (N = 101) (Figure 3). All CCs were named after putative founders of STs. The most numerous complex, CC650, comprised 20 STs (N = 89) originating from the three environments, including the most frequent STs ST222 (N = 27), ST625 (N = 19), and ST624 (N = 13). The second clonal complex, CC223, included seven STs (N = 16) also from the three habitats. The CC658–668 complex, the two putative founder, included 4 STs (N = 6). The other 16 CCs, comprised either three or two STs mostly originating from one environment.

**Figure 4:**
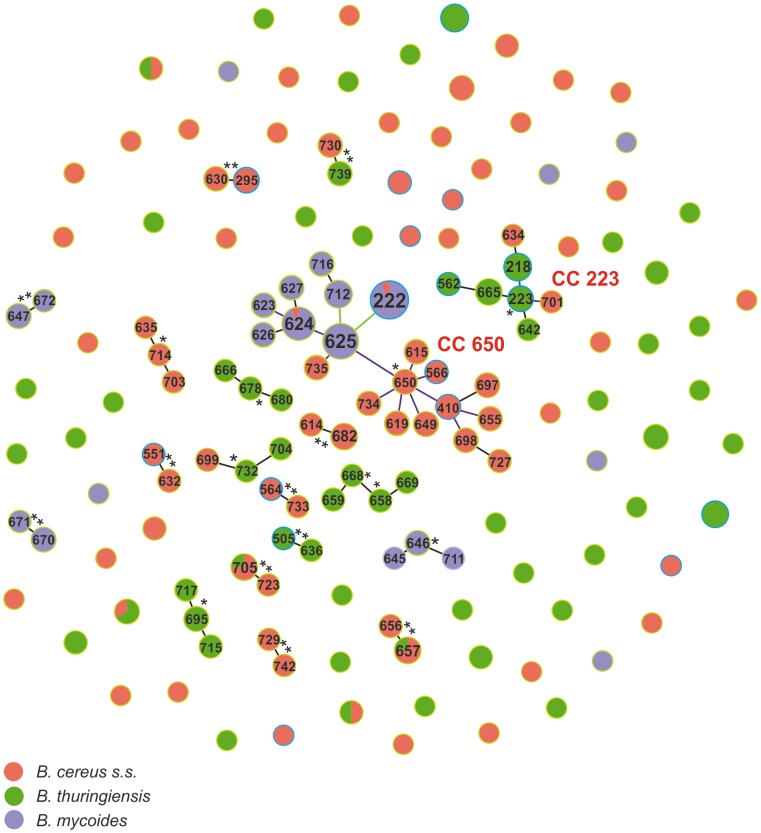
geoBURST analysis showing the clonal assignment of the STs present in soil isolates of *B. cereus*, *B. thuringiensis*, and *B. mycoides* originating from northeastern Poland. The CCs are named based on the ST assigned as a founder genotype (marked with a star) of the complex. The relative size of the circles indicates their prevalence among the *B. cereus s.l.* isolates. New STs characterized in this study are accentuated by a green halo, while STs present in the MLST database are accentuated by a blue halo.

In general, particular clonal complexes were composed of bacilli classified as the same species (Figure 4). Even in the most numerous complex, CC650 and CC223, a similar tendency was observed. In CC650, 11 STs consisting only of *B. cereus* (N = 20) composed one subcomplex around ST650, while nine STs with 65 *B. mycoides* and four *B. cereus* created the second subcomplex. The CC223 complex included 14 *B. thuringiensis* from all environments and only two *B. cereus* from Białowieża NP. The next two complexes, CC705–723 and CC656–657, comprised *B. cereus* and single *B. thuringiensis* of the same allelic profiles. CC732 and CC730–739 consisted of two STs with either *B. cereus* or *B. thuringiensis*.

Considering origins, eight CCs (N = 56) and 33 singletons (N = 37) were found among the forest isolates, nine CCs (N = 55) and 31 singletons (N = 40) in the marsh isolates, and four CCs (N = 48) and 25 singletons (N = 37) in the farm isolates (Figure S1). On the other hand, with regard to species classification, nine CCs (N = 52) and 44 singletons (N = 50) or six CCs (N = 34) and 40 singletons (N = 53), respectively, were observed among *B. cereus* and *B. thuringiensis*, while *B. mycoides* were classified only in four CCs (N = 78) and six singletons (N = 6) (Figure S2). It is worthy to note that ∼77% of *B. mycoides* belonged to CC650. According to the χ^2^ test, the proportion of the isolates forming clonal complexes within *B. mycoides* (78/84) differed significantly from the corresponding proportions within *B. cereus* (55/105) and *B. thuringiensis* (34/88); p-value of the test were <0.000 in both cases. However, the proportions in the latter two species did not differ significantly (p-value = 0.821).

The clonality of the isolates and the degree of association between alleles were assessed with the standardized index of association (I^S^_A_). The index for all 273 *B. cereus s.l.* isolates was calculated to be 0.477 (P<0.000). The separate calculation for each environment were I^S^_A_ = 0.364 (P<0.000) for the forest isolates, I^S^_A_ = 0.651 (P<0.000) for the marsh *B. cereus s.l.*, and I^S^_A_ = 0.476 (P<0.000) for the farm isolates. Among particular species, the I^S^_A_ indices were 0.452 (P = 0.000), 0.390 (P<0.000), and 0.520 (P = 0.000) for *B. cereus s.s.*, *B. thuringiensis*, and *B. mycoides*, respectively.

### Phylogenetic analysis

**Figure 5:**
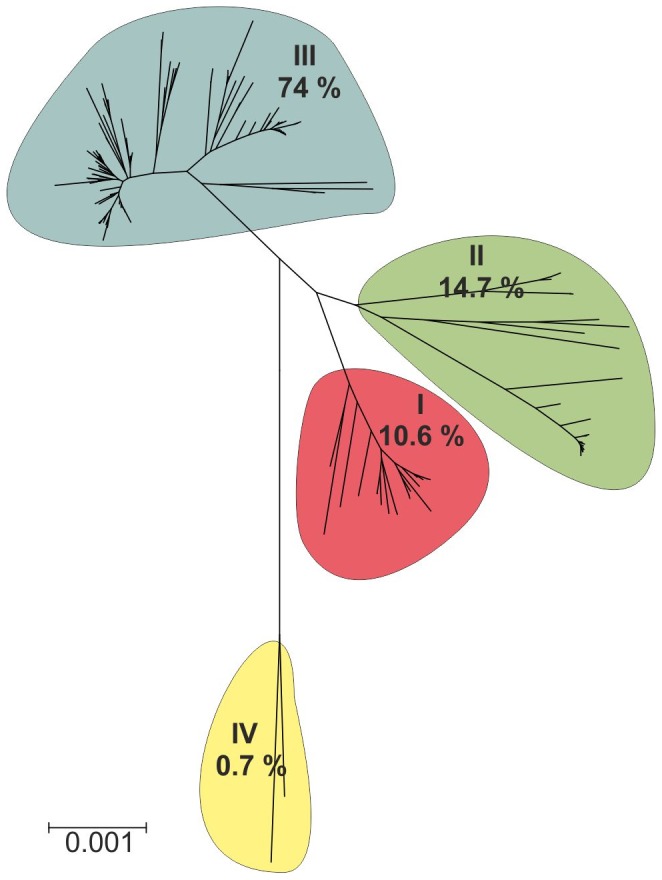
Phylogenetic tree of 273 *B. cereus s.l.* environmental isolates from northeastern Poland. The tree was constructed based on seven concatenated housekeeping loci (*glpF*, *gmk*, *ilvD*, *pta*, *pur*, *pycA*, and *tpi*) representing a total of 2,829 nucleotides, using the Neighbor-Joining (NJ) method implemented in MEGA4 software. Branch quality was evaluated using 1,000 replicates bootstraps [44]. The *B. cereus s.l.* isolates originated from northeastern Poland were separated into four major clades containing 10.6% (Clade I), 14.7% (Clade II), 74.0% (Clade III), and 0.7% (Clade IV) of environmental isolates. The pathogenic *B. cereus s.l.* references were clustered mostly with the minority of the environmental isolates of the clades I and II, whereas the environmental references were grouped together with the bacilli isolated in this study classified as the clade III.

The multiple alignment of 148 unique MLST concatenated sequences (2,829 bases long) of the environmental *B. cereus s.l.* and 28 reference sequences available at the MLST *B. cereus* database, revealed four major strain groups, defined according to Priest et al. (2004), as clade I, II, III and IV (Figure 5). Clade I contained approximately 11% of the environmental isolates (one *B. thuringiensis* and 28 *B. cereus*) mostly clustered with *B. anthracis* Ames (ST1), *B. cereus* ATCC 10987 (ST32), an atypical xylose-positive strain isolated from cheese, and *B. thuringiensis* HD868 (ST104). Based on the PubMLST dataset, this clade also contained strains isolated from clinical samples (ST123, ST133, ST141), food (ST123, ST152) and environment (ST309, ST152). Clade II containing approximately 15% of all environmental bacilli (28 *B. thuringiensis* and 11 *B. cereus*) clustered together with *B. cereus* type strain ATCC 14579 (ST4), *B. cereus* ATCC 11778 (ST34), and *B. thuringiensis* HD1 (ST10), HD12 (ST23), and HD73 (ST8), the lepidopteran-active strains. This clade also contained isolates from clinical samples (ST72, ST85), food (ST43) or natural environment (ST223, ST278, ST315). Clade III consisting of 74% of the bacteria isolated in this study grouped with *B. cereus s.l.* references designated as environmental isolates (ST286, ST305, ST332) in the MLST database, *B. weihenstephanensis* WSBC 10364 (ST42) and DSMZ 11821 (ST447), as well as *B. mycoides* ATCC 6462 (ST116). Clade IV contained only two *B. mycoides* from Jasienowka and *B. pseudomycoides* DSMZ 12442 (ST83).

**Figure 6:**
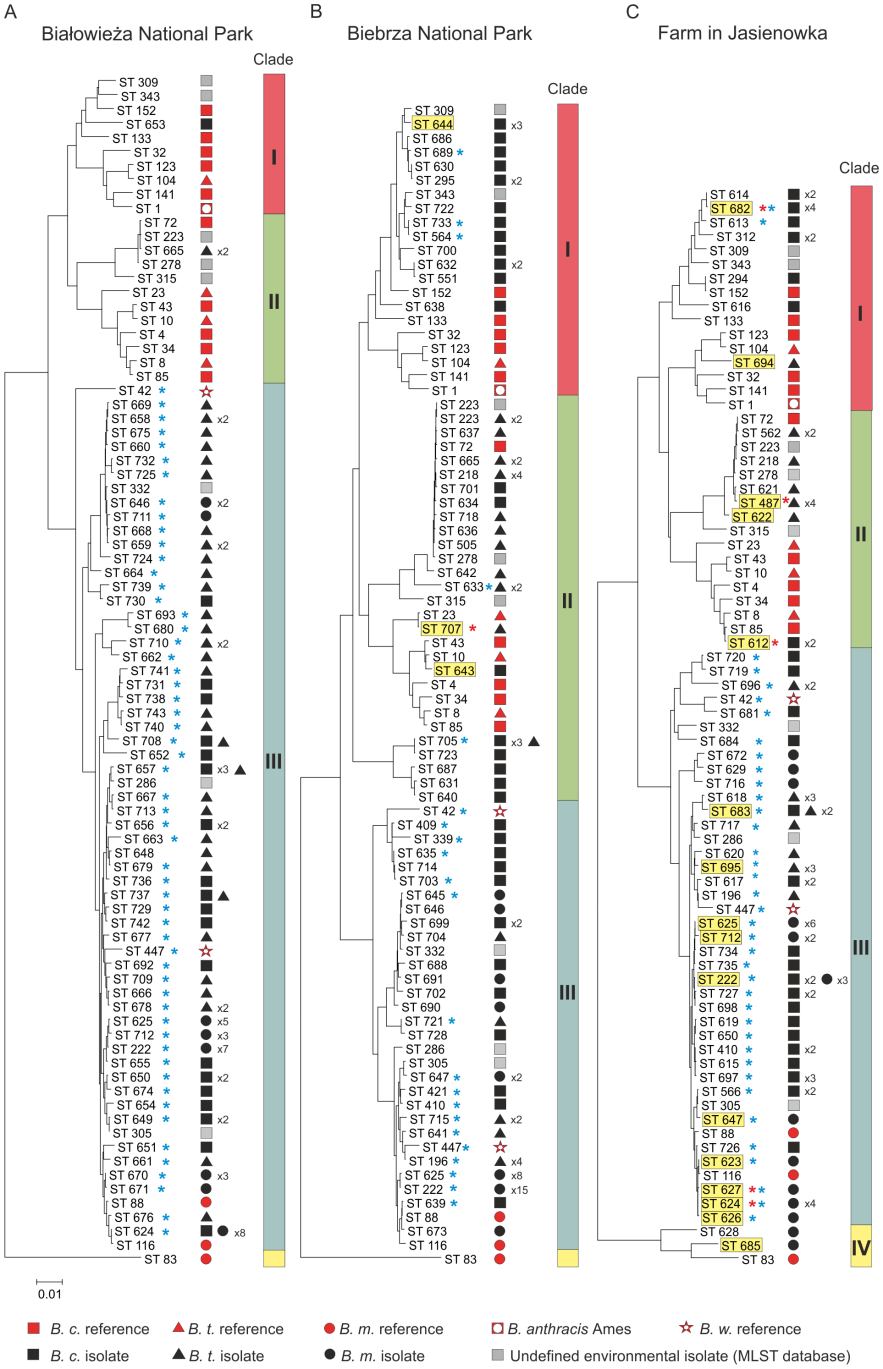
NJ phylogenic trees of the *B. cereus s.l.* environmental isolates originated from three locations in northeastern Poland and 28 reference strains. The trees prepared separately for each population were constructed as given in Figure 5. The isolates originated from Białowieża and Biebrza National Parks pertained to three clades, while those isolated from the farm samples clustered to four clades, with only two isolates in clade IV containing *B. pseudomycoides* reference strains. Detailed information on the isolates and the reference strains used in phylogenetic trees are given in Table S3. Yellow box indicates the *cytK* positive isolates. Red asterisk indicates the isolates that express the *cytK* gene in Real-Time PCR analysis. Blue asterisk indicates isolates able to grow at 7°C. *B.c.*, *B. cereus s.s.*; *B.t.*, *B. thuringiensis*; *B.m.*, *B. mycoides*, *B.a.*, *B. anthracis*, *B.w.*, *B. weihenstephanensis*.

Analysis of the sample collection sites and the phylogenetic tree showed a strong correlation between the clade designation and origin of isolates (Figure 6). Almost all *B. cereus s.l.* (97%) recovered from Białowieża NP clustered in clade III, while only one and two isolates from this habitat clustered with the references in clade II and III. Similarly, the majority (71%) of bacteria isolated from the farm clustered within clade III, and only 14 and 13% of isolates from this habitat were found in clade I and II. The bacilli originating from Biebrza NP clustered in clade I, II, and III in proportions of 17, 28, and 55%, respectively.

### Detection of toxin genes

The *cytK* gene was present in four isolates from Biebrza NP (three *B. cereus*, and one *B. thuringiensis*) and in 29 from the farm (five *B. cereus*, five *B. thuringiensis*, and 19 *B. mycoides*) (Figure 6, Table S4). However, based on Real-Time PCR data, the expression of the *cytK* gene was established for eight isolates (one and seven from the Biebrza NP and the farm, respectively), identified as *B. cereus* (N = 4), *B. thuringiensis* (N = 2), and *B. mycoides* (N = 2) (Figure 6). The relative expression varied from 0.55 (*B. mycoides* 35/1 from the farmland, ST627) to 1.02 (*B. thuringiensis* 56/3 from Biebrza NP, ST707) (Table S4).

The entomopathogenic properties of *B. thuringiensis* were assessed based on the presence of genes encoding δ-endotoxins. The *cry1* gene was found among 38, 24 and 22 bacilli from Białowieża NP, Biebrza NP and the farm, respectively, and four isolates (BPN 40/2, ST710; BPN 43/2, ST680; BPN 51/1, ST667; BPN 54/2, ST668) from Białowieża NP were also *cry2*-positive. Only *B. thuringiensis* BB 48/1 (ST721) and BB 56/3 (ST707) isolated from Biebrza NP, and *B. thuringiensis* JAS 10/2 (ST694) from the farmland harbored *cry4*. Interestingly, based on the phylogenetic trees (Figure 6) the BB 56/3 isolate (ST707) appeared to be closely related to *B. thuringiensis* HD12 (ST23), and JAS 10/2 (ST694) to HD868 (ST104).

### Temperature adaptation

Altogether, 189 environmental *B. cereus s.l.* isolates (69.2%) were able to grow at low temperature (Table S4). The majority of these isolates (95.8%) belonged to clade III (Figure 6). The largest number of isolates with psychrotrophic properties was found among bacilli isolated from Białowieża NP (95.7%), while growth at 7°C was observed only among 43.2 and 69.4% isolates from Biebrza NP and farmland, respectively. None of the isolates grew at 50°C.

## Discussion

In this report we present various aspects of the genetic structure and phylogeny of *B. cereus s.l.* isolates originating from three highly diverse habitats in northeastern Poland. In general, the density of these bacilli in “organic soil” was approximately twice as high when compared to “mineral soil”. Evidently these differences correlated with the concentration of nutrients and biogenic elements as suggested by Broughton et al. [45]. *B. cereus s.l.* isolated in this study demonstrated a high genetic diversity, but similar in all populations, as measured in MLST by the number of polymorphic sites, alleles, and sequence types. Heterogeneous habitats, such as soil, even with low concentration of nutrients as noted in the farm sampling, facilitates bacterial genetic divergence [46]. Zwick et al. [47] suggested that genes associated with metabolic processes under selection offer ecological specialization and may result from adaptation to specific deficiencies in the environment. Based on the *r/m* ratio, the diversity observed within the studied populations resulted mostly from mutation events, rather than from recombination events. Yet, some differences were noted at the level of recombination when forest bacilli (*r/m*: 1.2–2.7) were compared with marsh (*r/m*: 0.8–1.9) and farm (*r/m*: 0.8–1.8) populations. Thus, it is more likely that environmental conditions may influence recombination rates. *B. cereus s.l.* isolated in northeastern Poland undergo recombination slower than those tested by Didelot and Falush [39] (*r/m*: 1.3–2.8) or Didelot et al. [48] (*r/m*: 1.08–1.57). A comprehensive study by Vos and Didelot [49] comparing homologous recombination rates in bacteria and archaea showed significant variation from species to species, with the *r/m* ratio from 0.02 for *Leptospira interrogans* to even 63.3 for *Flavobacterium psychrophillum*. Apparently, recombination rates are not only influenced by bacterial lifestyle (pathogenic, commensal or mutualistic), but also by environmental conditions. Nevertheless, it is necessary to emphasize the impact of horizontal gene transfer (HGT) on bacterial diversity, particularly mediated by conjugation, which occurs especially in soil rich in organic material [50], [51].

The relatively large samples of *B. cereus s.l.* from three different ecologic habitats, allowed testing the hypothesis that specific genotypes of these bacilli occur in a particular natural habitat. This assumption was supported by the presence of 137 (92%) STs in bacilli originating from one specific habitat. Moreover, the description of new STs for 77% of the isolates provided additional evidence for the existence of specific genotypes within natural *B. cereus s.l.* populations. Owing to high dispersal ability of *Bacillus* spp. [21], the presence of ecologically distinct genotypes within environmental *B. cereus s.l.* populations must be based on adaptations to habitats, not on accidental colonization. In this regard, of particular importance is the statistically significant abundance of new genotypes within *B. cereus s.l.* originated from Białowieża NP when compared with those from Biebrza NP and the farm. The protection of Białowieża NP [52] appears to have an impact on the heterogeneity of ecological “microniches”, which affects the diversification of *B. cereus s.l.* into genetically different lineages. Nevertheless, it cannot be excluded that the forest environment itself may select for distinct ecotypes. Yet, the incidence of 11 STs (8%) simultaneously in more than one habitat is, to a minor extent, in contradiction to the hypothesis about association between bacterial genotypes and ecological niches. The latter STs probably represent polyphyletic genotypes persisting in a variety of environmental habitats, similarly as noted for *B. thuringiensis* HD73 (ST8) [28]. Thus, some genotypes within environmental *B. cereus s.l.* populations do not seem to be niche specialists. This observation probably also applies to other microorganisms in natural population. For instance Hunt et al. [53] noted remarkable narrow preferences within some *Vibrio* spp. genotypes, whereas others were broadly distributed in marine ecosystem.

In contrast to the diversity, the I^S^_A_ index, significantly different from zero, inferred a clonal population structure. It is generally accepted, that in clonal bacterial populations the genetic diversity is purged by selective sweeps [54]. Recombination in *Bacillus* spp. is unlikely to be frequent enough to prevent periodic selection events from occurring [54]. Indeed, the selective pressure on the alleles among the bacilli studied, as measured by *dN*/*dS* ratio, indicates a negative selection. Nevertheless, our data highlight the ecological diversity among very close relatives (e.g. within group III), which indicates that a selective sweep would not be expected to purge the diversity within an entire species. For instance, goeBURST grouped 172 bacilli isolated in this study into 19 complexes corresponding to bacterial clones, and 101 bacteria into 80 singletons. Thus, although the *dN*/*dS* and I^S^_A_ indices suggested clonal population structures, dynamic genetic diversification exists in natural populations of *B. cereus s.l.*, presumably allowing these bacteria to adapt to different ecological niches, and thereby increasing the number of ecologically distinct subpopulations, so called ecotypes [55]. In some cases ecotypes are identified as DNA sequence lineages, but more often an ecotype can encompass distinct evolutionary lineages [54]. In this study we found lineages of isolates, mostly within clade III, capable of growth at low temperature (thermal ecotypes), as previously observed among *B. cereus s.l.* and *B. thuringiensis* [30], [56]. However, in contrast to the previous report [30], in which we found association between the cytotoxic potential with some *B. thuringiensis* ecotypes, in this study the presence of *cytK* was mainly associated with isolates from farm samplings where human activity is extensive, while the gene was absent in isolates originating from Białowieża NP. Moreover, the *cytK* presence was intermixed among the lineages and species. This supports the opportunistic pathogenicity model of *B. cereus s.l.*, where the potential or ability to cause various diseases have no association with specific pathotypes [57]. The presence of *cytK* among *B. cereus s.s.*, *B. thuringiensis* and *B. mycoides*, indicate that food poisoning potential is not associated with species affiliation as suggested by Guinebretière et al. [57]. From an ecological point of view, it could be considered that toxin production is considerably “expensive” for the bacterial host, and presumably toxigenic genes would not be maintained if harboring them were disadvantageous [2]. Nevertheless, the problem of *B. cereus s.l.* pathogenicity and its relation to ecology of the group seems to be more complex and needs further investigation.

In phylogenetic analyses, only a small number of isolates from the marsh and farm samples clustered in clade I, a clade composed primarily of pathogenic bacilli [13]. These bacilli formed singletons and low-numbered complexes, which may suggest they are “atypical” environmental isolates. The part of STs forming clade III, created the CC650 complex (N = 89/202) and were classified as *B. cereus s.s.* and *B. mycoides*, whereas, clade II STs forming CC223 (N = 16/40) were mostly *B. thuringiensis* and *B. cereus s.s.* These two clonal complexes seem to be representative for these clades, and indicate their adaptation to specific niches, i.e., CC650 - soil matrices, and CC223 - insect larvae. Some taxonomists, based on studies of mostly human-associated isolates, disagree with the separation of *B. cereus s.l.* into distinct species [12], [13]. Hence, we hypothesized that the environmental *B. cereus s.l.* are also genetically highly related and should be classified to one species. However, the above hypothesis was not confirmed in our study as (i) only seven STs included bacilli classified into different species, (ii) the isolates classified to the same species but originated from different habitats exhibited a tendency of clustering to particular complexes, and (iii) *B. mycoides* isolates showed higher genetic similarity than *B. cereus s.s.* and *B. thuringiensis* confirmed statistically by significantly lower number of clonal complexes with high number of isolates (4 CCs; N = 78/84). The previous reports largely focused on *B. cereus s.s.*, *B. anthracis*, and/or *B. thuringiensis* [12], [28], [30]. Here we present for the first time the genetic structure for *B. mycoides*. Indeed this bacterium has no proven medical and/or economic significance, but it could alter bacterial populations dynamics, for instance by aiding other bacteria to adapt to specific environments or by facilitating horizontal gene transfer, as has been described for *B. cereus* and *B. thuringiensis* [28]. Although the idea of classification of the *B. cereus s.l.* isolates into one species has many proponents [12], [13], [18], this issue is still unresolved, especially as it relates to environmental isolates of the group. It seems that in contrast to the human-associated *B. cereus s.l.* exhibiting high levels of similarities [12], the environmental isolates are more intricate. We propose dividing *B. cereus s.l.* into two groups, the first which contains environmental isolates, and the second composed of those that may be clinically significant.

In summary, although high genetic diversity measured with MLST was observed among natural *B. cereus s.l.* isolates from three varied habitats in northeastern Poland, the populations appear to be clonal in nature. Nevertheless, the bacilli undergo dynamic genetic diversification, mostly resulting from mutation events. Further, a significantly high number of genotypes found within *B. cereus s.l.* populations are habitat-specific. In phylogenetic analyses described in this report, only a small number of isolates, mostly from environments associated with the anthropogenic factors (the Jasienowka farm and the Biebrza NP) clustered in clade I and were associated with pathogenic *B. cereus s.l.*, while the majority of isolates clustered in clade III comprising environmental bacilli from the MLST database. Based on genetic properties of the isolates we did not find strong arguments for merging of the particular species into one taxon. It seems that in contrast to human-associated *B. cereus s.l.*, which exhibit significant similarity, the environmental isolates are more complex.

## Supporting Information

Table S1 **The diagnostic feature used for species-level classification of the** ***B. cereus s.l.*** **isolates.**
(DOCX)

Table S2 **Primers used in this study.**
(DOCX)

Table S3 **Genetic diversity in the seven housekeeping loci within** ***B. cereus s.s.*** **,** ***B. thuringiensis*** **, and** ***B. mycoides*** **originated from northeastern Poland.**
(DOCX)

Table S4 **Characterization of the** ***B. cereus s.l.*** **isolates originated from Białowieża National Park (BPN), Biebrza National Park (BB), and the Jasienowka farm (JAS), and the 12 reference strains used in the phylogenetic analysis.**
(DOCX)

Figure S1 **geoBURST analysis.** The figures are showing the clonal assignment of the STs present in *B. cereus s.l.* originating from Białowieża National Park (A), Biebrza National Park (B), and the Jasienowka farm (C) in northeastern Poland. The CCs are named based on the ST assigned as a founder genotype (marked with a star) of the complex. The relative size of the circles indicates their prevalence among the *B. cereus s.l.* isolates. New STs characterized in this study are accentuated by a green halo, while STs present in the MLST database are accentuated by a blue halo.
(TIF)

Figure S2 **geoBURST analysis.** The figures are showing the clonal assignment of the STs present in soil isolates of *B. cereus s.s.* (A), *B. thuringiensis* (B), and *B. mycoides* (C) originating from northeastern Poland. The CCs are named based on the ST assigned as a founder genotype (marked with a star) of the complex. The relative size of the circles indicates their prevalence among the *B. cereus s.l.* isolates. New STs characterized in this study are accentuated by a green halo, while STs present in the MLST database are accentuated by a blue halo.
(TIF)
