# Supplementary material for: Eco-Genetic Structure of Bacillus cereus sensu lato Populations from Different Environments in Northeastern Poland
Source: PLoS One. 2013 Dec 2;8(12):e80175. doi: 10.1371/journal.pone.0080175 (PMC3846478; doi:10.1371/journal.pone.0080175)
Supplement: Table S1 — The diagnostic feature used for species-level classification of the B. cereus s.l. isolates. (DOCX) [file pone.0080175.s001.docx]

Table S1. The diagnostic feature used for species-level classification of the *B. cereus s.l.* isolates.

| **Diagnostic** | ***B. cereus*** | ***B. thuringiensis*** | ***B. anthracis*** | ***B. mycoides/*** | ***B. weihen-*** | ***B. cytotoxicus*** |
| --- | --- | --- | --- | --- | --- | --- |
| **feature** |  |  |  | ***B. pseudomycoides*** | ***stephanensis*** |  |
| Parasporal crystal synthesis | no | yes | no | no | no | no |
| Rhizoidal growth | no | no | no | yes | no | no |
| Sheep blood hemolysis | yes | yes | no | yes | yes | yes |
| Growth at 7 ^o^C | no | variable | no | no | yes | no |
| Growth at 50 ^o^C | no | no | no | no | no | yes |
