# Supplementary material for: Eco-Genetic Structure of Bacillus cereus sensu lato Populations from Different Environments in Northeastern Poland
Source: PLoS One. 2013 Dec 2;8(12):e80175. doi: 10.1371/journal.pone.0080175 (PMC3846478; doi:10.1371/journal.pone.0080175)
Supplement: Table S2 — Primers used in this study. (DOCX) [file pone.0080175.s002.docx]

Table S2. Primers used in this study.

| **Target** | **Primer** | **Nucleotide** | **Product** | **Reference** |
| --- | --- | --- | --- | --- |
| **gene** | **name** |  | **size (bp)** |  |
| **Primers for the δ-endotoxin genes** | | | | |
| *cry1* | Un1(d) | 5’-CATGATTCATGCGGCAGATAAAC-3’ | 277 | Ben-Dov et al., 1997 |
|  | Un1(r) | 5’-TTGTGACACTTCTGCTTCCCATT-3’ |  |  |
| *cry2* | Un2(d) | 5’-GTTATTCTTAATGCAGATGAATGGG-3’ | 701 | Ben-Dov et al., 1997 |
|  | Un2(r) | 5’-CGGATAAAATAATCTGGGAAATAGT-3’ |  |  |
| *cry3* | Un3(d) | 5’-CGTTATCGCAGAGAGATGACATTAAC-3’ | 589 | Ben-Dov et al., 1997 |
|  | Un3(r) | 5’-CATCTGTTGTTTCTGGAGGCAAT-3’ |  |  |
| *cry4* | Un4(d) | 5’-GCATATGATGTAGCGAAACAAGCC-3’ | 439 | Ben-Dov et al., 1997 |
|  | Un4(r) | 5’-GCGTGACATACCCATTTCCAGGTCC-3’ |  |  |
| *cry7,8* | Un7,8(d) | 5’-AAGCAGTGAATGCCTTGTTTAC-3’ | 420 | Ben-Dov et al., 1997 |
|  | Un7,8(r) | 5’-CTTCTAAACCTTGACTACTT-3’ |  |  |
| *cry9* | Un9(d) | 5’-CGGTGTTACTATTAGCGAGGGCGG-3’ | 354 | Ben-Dov et al., 1999 |
|  | Un9(r) | 5’-GTTTGAGCCGCTTCACAGCAATCC-3’ |  |  |
| **Primers for the cytK gene** | | | | |
| *cytK* | CytKF | 5’-GATAATATGACAATGTCTTTAAA-3’ | 1,011 | Swiecicka and Mahillon, 2006 |
| (PCR) | CytKR | 5’-GGAGAGAAACCGCTATTTGT-3’ |  |  |
| *cytK* | CytK(RT)-F | 5’-GGCCATTAGGCGTTACAGAA-3’ | 180 | M. Bartoszewicz (personal com.), 2013 |
| (RealTime PCR) | CytK(RT)-R | 5’-CTGGCGCTAGTGCAACATTA-3’ |  |  |
| *udp* | udpF | 5′-ACTAGAGAAACTTGGAAATGATCG-3′ | 101 | Reiter et al., 2011 |
|  | udpR | 5′-GACGCTTAATTGCACGGAAC-3′ |  |  |
| **Primers used in MLST analysis** | | | | |
| *glpF* | glpF_F | 5’-GCGTTTGTGCTGGTGTAAGT-3’ | 548 | www.pubmlst.org/bcereus/info/primers.shtml |
|  | glpF_R | 5’-CTGCAATCGGAAGGAAGAAG-3’ |  |  |
| *gmk* | gmk_F | 5’-ATTTAAGTGAGGAAGGGTAGG-3’ | 599 | www.pubmlst.org/bcereus/info/primers.shtml |
|  | gmk_R | 5’-GCAATGTTCACCAACCACAA-3’ |  |  |
| *ilvD* | ilvD_F | 5’-CGGGGCAAACATTAAGAGAA-3’ | 553 | www.pubmlst.org/bcereus/info/primers.shtml |
|  | ilvD_R | 5’-GGTTCTGGTCGTTTCCATTC-3’ |  |  |
|  | ilvD4_F | 5’-GCAGAGATTAAAGATAAGGA-3’ | 568 | www.pubmlst.org/bcereus/info/primers.shtml |
|  | ilvD2_R | 5’-GTTACCATTTGTGCATAACGC-3’ |  |  |
| *pta* | pta_F | 5’-GCAGAGCGTTTAGCAAAAGAA-3’ | 575 | www.pubmlst.org/bcereus/info/primers.shtml |
|  | pta_R | 5’-TGCAATGCGAGTTGCTTCTA-3’ |  |  |
| *pur* | pur_F | 5’-CTGCTGCGAAAAATCACAAA-3’ | 534 | www.pubmlst.org/bcereus/info/primers.shtml |
|  | pur_R | 5’-CTCACGATTCGCTGCAATAA-5’ |  |  |
| *pycA* | pycA_F | 5’-GCGTTAGGTGGAAACGAAAG-3’ | 549 | www.pubmlst.org/bcereus/info/primers.shtml |
|  | pycA_R | 5’-CGCGTCCAAGTTTATGGAAT-3’ |  |  |
| *tpi* | tpi_F | 5’-GCCCAGTAGCACTTAGCGAC-3’ | 557 | www.pubmlst.org/bcereus/info/primers.shtml |
|  | tpi_R | 5’-CCGAAACCGTCAAGAATGAT-3’ |  |  |
