# Supplementary material for: Eco-Genetic Structure of Bacillus cereus sensu lato Populations from Different Environments in Northeastern Poland
Source: PLoS One. 2013 Dec 2;8(12):e80175. doi: 10.1371/journal.pone.0080175 (PMC3846478; doi:10.1371/journal.pone.0080175)
Supplement: Table S3 — Genetic diversity in the seven housekeeping loci within B. cereus s.s. , B. thuringiensis , and B. mycoides originated from northeastern Poland. (DOCX) [file pone.0080175.s003.docx]

Table S3. Genetic diversity in the seven housekeeping loci within *B. cereus*, *B. thuringiensis*, and *B. mycoides* originated from northeastern Poland.

|  | **Białowieża National Park** | | |  | | |  | | | | |
| --- | --- | --- | --- | --- | --- | --- | --- | --- | --- | --- | --- |
|  | *B. cereus* (N = 25; ST = 20) | | | *B. thuringiensis* (N = 38; ST = 33) | | | *B. mycoides* (N = 30; ST = 8) | | | | |
|  | **Polymorphic** | **Number** | **Index** | **Polymorphic** | **Number** | **Index** | **Polymorphic** | | **Number** | | **Index** |
| **Locus** | **sites^a^** | **of alleles^b^** | ***d*N/*d*S^c^** | **sites^a^** | **of alleles^b^** | ***d*N/*d*S^c^** | **sites^a^** | | **of alleles^b^** | | ***d*N/*d*S^c^** |
| *glpF* | 21 (5.6) | 11 (5) | 0.030 | 26 (7.0) | 15 (7) | 0.045 | 8 (2.2) | | 5 (1) | | 0.040 |
| *gmk* | 31 (6.2) | 6 (4) | 0.030 | 32 (6.3) | 9 (7) | 0.037 | 3 (0.6) | | 2 (0) | | 0.145 |
| *ilvD* | 43 (10.9) | 11 (6) | 0.016 | 68 (17.3) | 14 (9) | 0.016 | 14 (3.6) | | 4 (1) | | 0.023 |
| *pta* | 27 (6.5) | 13 (7) | 0.024 | 34 (8.2) | 18 (9) | 0.016 | 3 (0.7) | | 2 (0) | | 0.000 |
| *pur* | 41 (11.8) | 10 (4) | 0.014 | 48 (13.8) | 17 (11) | 0.028 | 16 (4.6) | | 5 (1) | | 0.062 |
| *pycA* | 43 (11.8) | 9 (3) | 0.047 | 44 (12.1) | 9 (4) | 0.019 | 20 (5.5) | | 3 (0) | | 0.041 |
| *tpi* | 10 (2.3) | 8 (3) | 0.095 | 11 (2.5) | 7 (2) | 0.060 | 2 (0.5) | | 2 (0) | | 0.000 |
|  | **Biebrza National Park** | | |  | | |  | | | | |
|  | *B. cereus* (N = 39; ST = 32) | | | *B. thuringiensis* (N = 26; ST = 16) | | | *B. mycoides* (N = 30; ST = 8) | | | | |
|  | **Polymorphic** | **Number** | **Index** | **Polymorphic** | **Number** | **Index** | **Polymorphic** | | **Number** | | **Index** |
| **Locus** | **sites^a^** | **of alleles^b^** | ***d*N/*d*S^c^** | **sites^a^** | **of alleles^b^** | ***d*N/*d*S^c^** | **sites^a^** | | **of alleles^b^** | | ***d*N/*d*S^c^** |
| *glpF* | 37 (9.9) | 18 (6) | 0.094 | 26 (7.0) | 9 (3) | 0.118 | 7 (1.9) | | 5 (1) | | 0.052 |
| *gmk* | 57 (11.3) | 14 (4) | 0.015 | 49 (9.7) | 8 (1) | 0.017 | 3 (0.6) | | 2 (0) | | 0.145 |
| *ilvD* | 91 (23.2) | 18 (10) | 0.018 | 79 (20.1) | 8 (3) | 0.018 | 16 (4.1) | | 5 (2) | | 0.016 |
| *pta* | 41 (9.9) | 19 (5) | 0.015 | 28 (6.8) | 7 (2) | 0.020 | 11 (2.7) | | 4 (2) | | 0.029 |
| *pur* | 57 (16.4) | 16 (6) | 0.013 | 50 (14.4) | 8 (3) | 0.013 | 16 (4.6) | | 7 (2) | | 0.056 |
| *pycA* | 66 (18.2) | 14 (4) | 0.031 | 54 (14.9) | 8 (3) | 0.023 | 20 (5.5) | | 3 (0) | | 0.041 |
| *tpi* | 25 (5.7) | 18 (8) | 0.086 | 12 (2.8) | 8 (0) | 0.042 | 2 (0.5) | | 2 (0) | | 0.000 |
|  | **Farmland in Jasienowka** | | |  | | |  | | | | |
|  | *B. cereus* (N = 38; ST = 25) | | | *B. thuringiensis* (N = 23; ST = 13) | | | *B. mycoides* (N = 24; ST = 13) | | | | |
|  | **Polymorphic** | **Number** | **Index** | **Polymorphic** | **Number** | **Index** | **Polymorphic** | **Number** | | **Index** | |
| **Locus** | **sites^a^** | **of alleles^b^** | ***d*N/*d*S^c^** | **sites^a^** | **of alleles^b^** | ***d*N/*d*S^c^** | **sites^a^** | **of alleles^b^** | | ***d*N/*d*S^c^** | |
| *glpF* | 28 (7.5) | 15 (2) | 0.124 | 24 (6.5) | 9 (0) | 0.121 | 15 (4.0) | 5 (1) | | 0.122 | |
| *gmk* | 45 (8.9) | 14 (5) | 0.013 | 38 (7.5) | 6 (2) | 0.021 | 53 (10.5) | 3 (0) | | 0.027 | |
| *ilvD* | 77 (19.6) | 11 (3) | 0.014 | 62 (15.8) | 8 (2) | 0.019 | 66 (16.8) | 4 (1) | | 0.018 | |
| *pta* | 29 (7.0) | 10 (2) | 0.021 | 25 (6.0) | 10 (2) | 0.039 | 35 (8.5) | 5 (1) | | 0.018 | |
| *pur* | 55 (15.8) | 13 (3) | 0.028 | 45 (12.9) | 8 (4) | 0.016 | 47 (13.5) | 6 (1) | | 0.014 | |
| *pycA* | 56 (15.4) | 13 (4) | 0.029 | 53 (14.6) | 7 (2) | 0.027 | 38 (10.5) | 4 (0) | | 0.026 | |
| *tpi* | 12 (2.8) | 9 (1) | 0.098 | 17 (3.9) | 6 (1) | 0.080 | 22 (5.1) | 4 (2) | | 0.052 | |

^a^ Number of polymorphic sites and percentage (in parentheses) calculated as the number of polymorphic sites divided by the length of the sequenced fragment.

^b^ Number of new alleles described in this study is given parentheses.

^c^ Ratio of nonsynonymous(*d*_N_) to synonymous (*d*_S_) substitutions per nucleotide site. *d*N/*d*S < 1 indicates that the loci is subjected to purifying selection.
