# Supplementary material for: Eco-Genetic Structure of Bacillus cereus sensu lato Populations from Different Environments in Northeastern Poland
Source: PLoS One. 2013 Dec 2;8(12):e80175. doi: 10.1371/journal.pone.0080175 (PMC3846478; doi:10.1371/journal.pone.0080175)
Supplement: Table S4 — Characterization of the B. cereus s.l. isolates originated from Białowieża National Park (BPN), Biebrza National Park (BB), and the Jasienowka farm (JAS), and the 12 reference strains used in the phylogenetic analysis. (DOCX) [file pone.0080175.s004.docx]

Table S4. Characterization of the *B. cereus s.l.* isolates originated from three habitats in northeastern Poland, and 12 reference strains used in the phylogenetic analysis.

| **Isolate^a^** | **Origin^b^** | **Growth** | ***cry*^d^** | ***cytK*^e^** | **ST^f^** | **Clade^g^** | **CC^h^** |
| --- | --- | --- | --- | --- | --- | --- | --- |
|  |  | **at 7^o^C^c^** |  |  |  |  |  |
| *B.c.* 33/4 | BPN |  |  |  | 653^N^ | I | S |
| *B.t*. 44/2 | BPN |  | + |  | 665^N^ | II | CC223 |
| *B.t*. 45/4 | BPN |  | + |  | 665^N^ | II | CC223 |
| *B.t*. 55/2 | BPN | + | + |  | 669^N^ | III | CC658-668 |
| *B.t.* 05/4 | BPN | + | + |  | 658^N^ | III | CC658-668 |
| *B.t.* 60/3 | BPN | + | + |  | 658^N^ | III | CC658-668 |
| *B.t*. 05/2 | BPN | + | + |  | 675^N^ | III | S |
| *B.t.* 10/2 | BPN | + | + |  | 660^N^ | III | S |
| *B.t.* 59/2 | BPN | + | + |  | 732^N^ | III | CC732 |
| *B.t*. 50/1 | BPN | + | + |  | 725^N^ | III | S |
| *B.m*. 06/1 | BPN | + |  |  | 646^N^ | III | CC646 |
| *B.m*. 42/1 | BPN | + |  |  | 646^N^ | III | CC646 |
| *B.m*. 03/1 | BPN | + |  |  | 711^N^ | III | CC646 |
| *B.t.* 54/2 | BPN | + | +, +^2^ |  | 668^N^ | III | CC658-668 |
| *B.t*. 07/3 | BPN | + | + |  | 659^N^ | III | CC658-668 |
| *B.t*. 56/1 | BPN | + | + |  | 659^N^ | III | CC658-668 |
| *B.t.* 05/3 | BPN | + | + |  | 724^N^ | III | S |
| *B.t.* 42/2 | BPN | + | + |  | 664^N^ | III | S |
| *B.t.* 57/2 | BPN | + | + |  | 739^N^ | III | CC730-739 |
| *B.c*. 42/1 | BPN | + |  |  | 730^N^ | III | CC730-739 |
| *B.t*. 53/3 | BPN | + | + |  | 693^N^ | III | S |
| *B.t*. 43/2 | BPN | + | +, +^2^ |  | 680^N^ | III | CC678 |
| *B.t.* 36/4 | BPN | + | + |  | 710^N^ | III | S |
| *B.t*. 40/2 | BPN | + | +, +^2^ |  | 710^N^ | III | S |
| *B.t*. 37/2 | BPN | + | + |  | 662^N^ | III | S |
| *B.t.* 41/1 | BPN | + | + |  | 741^N^ | III | S |
| *B.c.* 54/1 | BPN | + |  |  | 731^N^ | III | S |
| *B.c.* 57/3 | BPN | + |  |  | 738^N^ | III | S |
| *B.t*. 38/4 | BPN | + | + |  | 743^N^ | III | S |
| *B.t*. 57/4 | BPN | + | + |  | 740^N^ | III | S |
| *B.c.* 58/4 | BPN | + |  |  | 708^N^ | III | S |
| *B.t*. 03/1 | BPN | + | + |  | 708^N^ | III | S |
| *B.c.* 32/3 | BPN | + |  |  | 652^N^ | III | S |
| *B.c*. 50/3 | BPN | + |  |  | 657^N^ | III | CC656-657 |
| *B.c*. 52/2 | BPN | + |  |  | 657^N^ | III | CC656-657 |
| *B.c*. 56/3 | BPN | + |  |  | 657^N^ | III | CC656-657 |
| *B.t*. 30/3 | BPN | + | + |  | 657^N^ | III | CC656-657 |
| *B.t.* 51/1 | BPN | + | +, +^2^ |  | 667^N^ | III | S |
| *B.t.* 30/4 | BPN | + | + |  | 713^N^ | III | S |
| *B.c.* 43/4 | BPN | + |  |  | 656^N^ | III | CC656-657 |
| *B.c.* 55/4 | BPN | + |  |  | 656^N^ | III | CC656-657 |
| *B.t.* 40/1 | BPN | + | + |  | 663^N^ | III | S |
| *B.t.* 06/1 | BPN |  | + |  | 648^N^ | III | S |
| *B.t.* 35/2 | BPN | + | + |  | 679^N^ | III | S |
| *B.c*. 30/1 | BPN | + |  |  | 736^N^ | III | S |
| *B.c.* 34/4 | BPN | + |  |  | 737^N^ | III | S |
| *B.t*. 12/1 | BPN | + | + |  | 737^N^ | III | S |
| *B.c.* 21/1 | BPN | + |  |  | 729^N^ | III | CC729-742 |
| *B.c.* 36/3 | BPN | + |  |  | 742^N^ | III | CC729-742 |
| *B.t.* 51/2 | BPN | + | + |  | 677^N^ | III | S |
| *B.c*. 53/1 | BPN | + |  |  | 692^N^ | III | S |
| *B.t.* 02/2 | BPN | + | + |  | 709^N^ | III | S |
| *B.t.* 47/1 | BPN | + | + |  | 666^N^ | III | CC678 |
| *B.t.* 29/1 | BPN | + | + |  | 678^N^ | III | CC678 |
| *B.t.* 54/4 | BPN | + | + |  | 678^N^ | III | CC678 |
| *B.m*. 08/1 | BPN | + |  |  | 625^N^ | III | CC650 |
| *B.m*. 09/1 | BPN | + |  |  | 625^N^ | III | CC650 |
| *B.m*. 37/1 | BPN | + |  |  | 625^N^ | III | CC650 |
| *B.m*. 38/1 | BPN | + |  |  | 625^N^ | III | CC650 |
| *B.m*. 48/1 | BPN | + |  |  | 625^N^ | III | CC650 |
| *B.m.* 05/1 | BPN | + |  |  | 712^N^ | III | CC650 |
| *B.m*. 15/1 | BPN | + |  |  | 712^N^ | III | CC650 |
| *B.m.* 20/1 | BPN | + |  |  | 712^N^ | III | CC650 |
| *B.m*. 13/1 | BPN | + |  |  | 222 | III | CC650 |
| *B.m*. 16/1 | BPN | + |  |  | 222 | III | CC650 |
| *B.m*. 29/1 | BPN | + |  |  | 222 | III | CC650 |
| *B.m*. 32/1 | BPN | + |  |  | 222 | III | CC650 |
| *B.m*. 52/1 | BPN | + |  |  | 222 | III | CC650 |
| *B.m*. 56/1 | BPN | + |  |  | 222 | III | CC650 |
| *B.m*. 57/1 | BPN | + |  |  | 222 | III | CC650 |
| *B.c*. 37/1 | BPN | + |  |  | 655^N^ | III | CC650 |
| *B.c.* 13/1 | BPN | + |  |  | 650^N^ | III | CC650 |
| *B.c*. 36/2 | BPN | + |  |  | 650^N^ | III | CC650 |
| *B.c.* 38/2 | BPN | + |  |  | 674^N^ | III | S |
| *B.c.* 35/4 | BPN | + |  |  | 654^N^ | III | S |
| *B.c*. 08/1 | BPN | + |  |  | 649^N^ | III | CC650 |
| *B.c.* 08/4 | BPN | + |  |  | 649^N^ | III | CC650 |
| *B.c.* 28/4 | BPN | + |  |  | 651^N^ | III | S |
| *B.t.* 32/4 | BPN | + | + |  | 661^N^ | III | S |
| *B.m.* 01/1 | BPN | + |  |  | 670^N^ | III | CC670-671 |
| *B.m.* 04/1 | BPN | + |  |  | 670^N^ | III | CC670-671 |
| *B.m*. 28/1 | BPN | + |  |  | 670^N^ | III | CC670-671 |
| *B.m.* 53/1 | BPN | + |  |  | 671^N^ | III | CC670-671 |
| *B.t.* 23/2 | BPN | + | + |  | 676^N^ | III | S |
| *B.c*. 60/1 | BPN | + |  |  | 624^N^ | III | CC650 |
| *B.m*. 12/1 | BPN | + |  |  | 624^N^ | III | CC650 |
| *B.m*. 26/1 | BPN | + |  |  | 624^N^ | III | CC650 |
| *B.m*. 31/1 | BPN | + |  |  | 624^N^ | III | CC650 |
| *B.m*. 35/1 | BPN | + |  |  | 624^N^ | III | CC650 |
| *B.m*. 36/1 | BPN | + |  |  | 624^N^ | III | CC650 |
| *B.m*. 39/1 | BPN | + |  |  | 624^N^ | III | CC650 |
| *B.m*. 40/1 | BPN | + |  |  | 624^N^ | III | CC650 |
| *B.m*. 45/1 | BPN | + |  |  | 624^N^ | III | CC650 |
| *B.c.* 04/1 | BB |  |  |  | 644^N^ | I | S |
| *B.c.* 51/4 | BB |  |  | + | 644^N^ | I | S |
| *B.c*. 54/4 | BB |  |  | + | 644^N^ | I | S |
| *B.c.* 20/4 | BB |  |  |  | 686^N^ | I | S |
| *B.c.* 25/4 | BB | + |  |  | 689^N^ | I | S |
| *B.c.* 08/1 | BB |  |  |  | 630^N^ | I | CC295-630 |
| *B.c.* 01/3 | BB |  |  |  | 295 | I | CC295-630 |
| *B.c*. 54/3 | BB |  |  |  | 295 | I | CC295-630 |
| *B.c*. 01/1 | BB |  |  |  | 722^N^ | I | S |
| *B.c*. 18/1 | BB | + |  |  | 733^N^ | I | CC564-733 |
| *B.c.* 26/4 | BB | + |  |  | 564 | I | CC564-733 |
| *B.c*. 18/3 | BB |  |  |  | 700^N^ | I | S |
| *B.c.* 19/4 | BB |  |  |  | 632^N^ | I | CC551-632 |
| *B.c.* 50/5 | BB |  |  |  | 632^N^ | I | CC551-632 |
| *B.c*. 25/1 | BB |  |  |  | 551 | I | CC551-632 |
| *B.c.* 16/1 | BB |  |  |  | 638^N^ | I | S |
| *B.t.* 51/3 | BB |  | + |  | 223 | II | CC223 |
| *B.t.* 56/2 | BB |  | + |  | 223 | II | CC223 |
| *B.t.* 15/2 | BB |  | + |  | 637^N^ | II | S |
| *B.t.* 52/5 | BB |  | + |  | 665^N^ | II | CC223 |
| *B.t.* 58/1 | BB |  | + |  | 665^N^ | II | CC223 |
| *B.t.* 17/4 | BB |  | + |  | 218 | II | CC223 |
| *B.t.* 21/3 | BB |  | + |  | 218 | II | CC223 |
| *B.t.* 44/5 | BB |  | + |  | 218 | II | CC223 |
| *B.t.* 47/4 | BB |  | + |  | 218 | II | CC223 |
| *B.c.* 23/4 | BB |  |  |  | 701^N^ | II | CC223 |
| *B.c*. 28/4 | BB |  |  |  | 634^N^ | II | CC223 |
| *B.t.* 48/5 | BB |  | + |  | 718^N^ | II | S |
| *B.t.* 10/5 | BB |  | + |  | 636^N^ | II | CC505-636 |
| *B.t*. 42/3 | BB |  | + |  | 505 | II | CC505-636 |
| *B.t.* 43/5 | BB |  | + |  | 642^N^ | II | CC223 |
| *B.t.*06/1 | BB | + | + |  | 633^N^ | II | S |
| *B.t.* 27/1 | BB |  | + |  | 633^N^ | II | S |
| *B.t.* 56/3 | BB |  | +^4^ | + (1.02) | 707^N^ | II | S |
| *B.c.* 45/2 | BB |  |  | + | 643^N^ | II | S |
| *B.c.* 01/2 | BB |  |  |  | 705^N^ | II | CC705-723 |
| *B.c.* 25/5 | BB | + |  |  | 705^N^ | II | CC705-723 |
| *B.c*. 34/5 | BB | + |  |  | 705^N^ | II | CC705-723 |
| *B.t.* 32/5 | BB |  | + |  | 705^N^ | II | CC705-723 |
| *B.c*. 16/2 | BB |  |  |  | 723^N^ | II | CC705-723 |
| *B.c.* 02/1 | BB |  |  |  | 687^N^ | II | S |
| *B.c*. 17/5 | BB |  |  |  | 631^N^ | II | S |
| *B.c.* 31/5 | BB |  |  |  | 640^N^ | II | S |
| *B.c.* 33/2 | BB | + |  |  | 409 | III | S |
| *B.c*. 22/2 | BB | + |  |  | 339 | III | S |
| *B.c*. 29/2 | BB | + |  |  | 635^N^ | III | CC714 |
| *B.c.* 02/3 | BB |  |  |  | 714^N^ | III | CC714 |
| *B.c.* 14/1 | BB | + |  |  | 703^N^ | III | CC714 |
| *B.m.* 10/1 | BB | + |  |  | 645^N^ | III | CC646 |
| *B.m*.57/1 | BB |  |  |  | 646^N^ | III | CC646 |
| *B.c.* 11/4 | BB |  |  |  | 699^N^ | III | CC732 |
| *B.c.* 14/5 | BB |  |  |  | 699^N^ | III | CC732 |
| *B.t.* 30/5 | BB |  | + |  | 704^N^ | III | CC732 |
| *B.c.* 05/3 | BB |  |  |  | 688^N^ | III | S |
| *B.m.* 48/1 | BB |  |  |  | 691^N^ | III | S |
| *B.c.* 12/1 | BB |  |  |  | 702^N^ | III | S |
| *B.m.* 46/1 | BB |  |  |  | 690^N^ | III | S |
| *B.t.* 48/1 | BB | + | +^4^ |  | 721^N^ | III | S |
| *B.c.* 53/3 | BB |  |  |  | 728^N^ | III | S |
| *B.m.* 33/1 | BB | + |  |  | 647^N^ | III | CC647-672 |
| *B.m.* 58/1 | BB | + |  |  | 647^N^ | III | CC647-672 |
| *B.c.* 52/3 | BB | + |  |  | 421 | III | S |
| *B.c.* 20/1 | BB | + |  |  | 410 | III | CC650 |
| *B.t.* 27/4 | BB | + | + |  | 715^N^ | III | CC695 |
| *B.t*. 46/2 | BB | + | + |  | 715^N^ | III | CC695 |
| *B.t.* 40/3 | BB | + | + |  | 641^N^ | III | S |
| *B.t.* 07/2 | BB |  | + |  | 196 | III | S |
| *B.t*. 15/1 | BB | + | + |  | 196 | III | S |
| *B.t*. 24/2 | BB | + | + |  | 196 | III | S |
| *B.t*. 58/3 | BB | + | + |  | 196 | III | S |
| *B.m.* 01/1 | BB | + |  |  | 625^N^ | III | CC650 |
| *B.m*. 02/1 | BB |  |  |  | 625^N^ | III | CC650 |
| *B.m*. 08/1 | BB |  |  |  | 625^N^ | III | CC650 |
| *B.m*. 14/1 | BB | + |  |  | 625^N^ | III | CC650 |
| *B.m*. 40/1 | BB | + |  |  | 625^N^ | III | CC650 |
| *B.m*. 51/1 | BB | + |  |  | 625^N^ | III | CC650 |
| *B.m.* 54/1 | BB | + |  |  | 625^N^ | III | CC650 |
| *B.m*. 55/1 | BB |  |  |  | 625^N^ | III | CC650 |
| *B.m*. 04/1 | BB | + |  |  | 222 | III | CC650 |
| *B.m*. 05/1 | BB | + |  |  | 222 | III | CC650 |
| *B.m*. 07/1 | BB |  |  |  | 222 | III | CC650 |
| *B.m.* 16/1 | BB | + |  |  | 222 | III | CC650 |
| *B.m.* 21/1 | BB | + |  |  | 222 | III | CC650 |
| *B.m.* 22/1 | BB |  |  |  | 222 | III | CC650 |
| *B.m.* 24/1 | BB | + |  |  | 222 | III | CC650 |
| *B.m*. 26/1 | BB | + |  |  | 222 | III | CC650 |
| *B.m.* 30/1 | BB | + |  |  | 222 | III | CC650 |
| *B.m*. 31/1 | BB | + |  |  | 222 | III | CC650 |
| *B.m.* 37/1 | BB | + |  |  | 222 | III | CC650 |
| *B.m.* 41/1 | BB | + |  |  | 222 | III | CC650 |
| *B.m.* 45/1 | BB | + |  |  | 222 | III | CC650 |
| *B.m.* 49/1 | BB | + |  |  | 222 | III | CC650 |
| *B.m.* 50/1 | BB | + |  |  | 222 | III | CC650 |
| *B.c*. 23/3 | BB | + |  |  | 639^N^ | III | S |
| *B.m.* 34/1 | BB |  |  |  | 673^N^ | III | S |
| *B.c*. 31/2 | JAS |  |  |  | 614^N^ | I | CC614-682 |
| *B.c*. 67/5 | JAS |  |  |  | 614^N^ | I | CC614-682 |
| *B.c*. 08/1 | JAS |  |  | + (0.90) | 682^N^ | I | CC614-682 |
| *B.c.* 11/1 | JAS |  |  |  | 682^N^ | I | CC614-682 |
| *B.c.* 23/4 | JAS |  |  | + (0,70) | 682^N^ | I | CC614-682 |
| *B.c.* 36/3 | JAS | + |  |  | 682^N^ | I | CC614-682 |
| *B.c.* 28/2 | JAS | + |  |  | 613^N^ | I | S |
| *B.c*. 05/4 | JAS |  |  |  | 312 | I | S |
| *B.c.* 07/5 | JAS |  |  |  | 312 | I | S |
| *B.c.* 90/2 | JAS |  |  |  | 294 | I | S |
| *B.c.* 92/2 | JAS |  |  |  | 616^N^ | I | S |
| *B.t.* 10/2 | JAS |  | +^4^ | + | 694^N^ | I | S |
| *B.t*. 03/3 | JAS |  | + |  | 562 | II | CC223 |
| *B.t.* 100/3 | JAS |  | + |  | 562 | II | CC223 |
| *B.t.* 22/1 | JAS |  | + |  | 218 | II | CC223 |
| *B.t.* 42/3 | JAS |  | + |  | 621^N^ | II | S |
| *B.t*. 04/4 | JAS |  | + |  | 487 | II | S |
| *B.t.* 09/5 | JAS |  | + |  | 487 | II | S |
| *B.t.* 13/1 | JAS |  | + |  | 487 | II | S |
| *B.t.* 32/4 | JAS |  | + | + (0,71) | 487 | II | S |
| *B.t.* 63/5 | JAS |  | + | + | 622^N^ | II | S |
| *B.c.* 24/2 | JAS |  |  | + (1.01) | 612^N^ | II | S |
| *B.c.* 35/2 | JAS |  |  | + (0.86) | 612^N^ | II | S |
| *B.c*. 74/1 | JAS | + |  |  | 720^N^ | III | S |
| *B.c*. 82/3 | JAS | + |  |  | 719^N^ | III | S |
| *B.t.* 30/2 | JAS | + | + |  | 696^N^ | III | S |
| *B.t*. 32/2 | JAS | + | + |  | 696^N^ | III | S |
| *B.c.* 01/4 | JAS | + |  |  | 681^N^ | III | S |
| *B.c*. 84/1 | JAS | + |  |  | 684^N^ | III | S |
| *B.m.* 100/1 | JAS | + |  |  | 672^N^ | III | CC647-672 |
| *B.m*. 91/1 | JAS | + |  |  | 629^N^ | III | S |
| *B.m.* 76/1 | JAS | + |  |  | 716^N^ | III | CC650 |
| *B.t.* 12/5 | JAS | + | + |  | 618^N^ | III | S |
| *B.t*. 58/5 | JAS | + | + |  | 618^N^ | III | S |
| *B.t*. 62/4 | JAS |  | + |  | 618^N^ | III | S |
| *B.c.* 80/5 | JAS | + |  |  | 683^N^ | III | S |
| *B.t*. 60/4 | JAS | + | + |  | 683^N^ | III | S |
| *B.t.* 83/2 | JAS | + | + | + | 683^N^ | III | S |
| *B.t.* 100/4 | JAS | + | + |  | 717^N^ | III | CC695 |
| *B.t*. 41/1 | JAS | + | + |  | 620^N^ | III | S |
| *B.t*. 05/5 | JAS | + | + |  | 695^N^ | III | CC695 |
| *B.t.* 21/4 | JAS | + | + |  | 695^N^ | III | CC695 |
| *B.t.* 78/1 | JAS | + | + | + | 695^N^ | III | CC695 |
| *B.c.* 06/3 | JAS | + |  |  | 617^N^ | III | S |
| *B.c.* 74/3 | JAS | + |  |  | 617^N^ | III | S |
| *B.t.* 98/4 | JAS | + | + |  | 196 | III | S |
| *B.m.* 20/1 | JAS | + |  | + | 625^N^ | III | CC650 |
| *B.m.* 23/1 | JAS | + |  | + | 625^N^ | III | CC650 |
| *B.m.* 27/1 | JAS | + |  | + | 625^N^ | III | CC650 |
| *B.m*. 39/1 | JAS | + |  | + | 625^N^ | III | CC650 |
| *B.m.* 58/1 | JAS | + |  | + | 625^N^ | III | CC650 |
| *B.m.* 81/1 | JAS | + |  | + | 625^N^ | III | CC650 |
| *B.m*. 07/1 | JAS | + |  |  | 712^N^ | III | CC650 |
| *B.m.* 30/1 | JAS | + |  | + | 712^N^ | III | CC650 |
| *B.c*. 78/2 | JAS | + |  |  | 734^N^ | III | CC650 |
| *B.c*. 30/3 | JAS | + |  |  | 735^N^ | III | CC650 |
| *B.c.* 06/1 | JAS | + |  | + | 222 | III | CC650 |
| *B.c.* 15/2 | JAS | + |  |  | 222 | III | CC650 |
| *B.m*. 17/1 | JAS | + |  | + | 222 | III | CC650 |
| *B.m*. 48/1 | JAS | + |  | + | 222 | III | CC650 |
| *B.m.* 62/1 | JAS | + |  | + | 222 | III | CC650 |
| *B.c.* 23/1 | JAS | + |  |  | 727^N^ | III | CC650 |
| *B.c*. 68/2 | JAS | + |  |  | 727^N^ | III | CC650 |
| *B.c.* 76/3 | JAS | + |  |  | 698^N^ | III | CC650 |
| *B.c.* 26/2 | JAS | + |  |  | 619^N^ | III | CC650 |
| *B.c.* 84/4 | JAS | + |  |  | 650^N^ | III | CC650 |
| *B.c.* 21/5 | JAS | + |  |  | 410 | III | CC650 |
| *B.c.* 85/1 | JAS | + |  |  | 410 | III | CC650 |
| *B.c*. 83/3 | JAS |  |  |  | 615^N^ | III | CC650 |
| *B.c*. 39/1 | JAS |  |  |  | 697^N^ | III | CC650 |
| *B.c.* 81/4 | JAS | + |  |  | 697^N^ | III | CC650 |
| *B.c*. 86/1 | JAS | + |  |  | 697^N^ | III | CC650 |
| *B.c.* 19/1 | JAS | + |  |  | 566 | III | CC650 |
| *B.c.* 21/1 | JAS | + |  |  | 566 | III | CC650 |
| *B.m.* 45/1 | JAS | + |  | + | 647^N^ | III | CC647-672 |
| *B.c.* 94/5 | JAS | + |  |  | 726^N^ | III | S |
| *B.m.* 09/1 | JAS | + |  | + | 623^N^ | III | CC650 |
| *B.m.* 35/1 | JAS | + |  | + (0.55) | 627^N^ | III | CC650 |
| *B.m*. 15/1 | JAS | + |  | + (0.76) | 624^N^ | III | CC650 |
| *B.m.* 41/1 | JAS | + |  | + | 624^N^ | III | CC650 |
| *B.m*. 60/1 | JAS | + |  | + | 624^N^ | III | CC650 |
| *B.m*. 82/1 | JAS | + |  | + | 624^N^ | III | CC650 |
| *B.m.* 21/1 | JAS | + |  | + | 626^N^ | III | CC650 |
| *B.m.* 72/1 | JAS |  |  |  | 628^N^ | IV | S |
| *B.m.* 51/1 | JAS |  |  | + | 685^N^ | IV | S |
| DSMZ 11821 | DSMZ | + |  |  | 447 | III | N.D. |
| DSMZ 12442 | DSMZ |  |  |  | 83 | IV | N.D. |
| WSBC 10364 | WSBC | + |  |  | 42 | III | N.D. |
| HD1 | BGSC |  | + | + (N.D.) | 10 | II | N.D. |
| HD12 | BGSC |  | + |  | 23 | II | N.D. |
| HD73 | BGSC |  | + | + (N.D.) | 8 | II | N.D. |
| HD868 | BGSC | N.D. | N.D. | N.D. | 104 | I | N.D. |
| ATCC 10987 | ATCC |  |  | + (N.D.) | 32 | I | N.D. |
| ATCC 14579 | ATCC |  |  | + (ref.) | 4 | II | N.D. |
| ATCC 11778 | ATCC | N.D. |  | N.D | 34 | II | N.D. |
| ATCC 6462 | ATCC | + |  | N.D. | 116 | III | N.D. |
| Ames | - | N.D. |  |  | 1 | I | N.D. |

^a^ *B.c.*, *B. cereus*; *B.t.*, *B. thuringiensis*; *B.m.*, *B. mycoides*; *B.a.*, *B. anthracis*; *B.w.*, *B. weihenstephanensis*; DSMZ 11821, *B. weihenstephanensis* type strain; DSMZ 12442, *B. pseudomycoides* DSMZ 12442; WSBC 10364, *B. weihenstephanensis* WSBC 10364; HD1, *B. thuringiensis* HD1; HD12, *B. thuringiensis* HD12; HD73, *B. thuringiensis* HD73; HD868, *B. thuringiensis* HD868; ATCC 10987, *B. cereus* ATCC 10987; ATCC 14579, *B. cereus* ATCC 14579; ATCC 11778, *B. cereus* ATCC 11778; ATCC 6462, *B. mycoides* ATCC 6462; Ames, *B. anthracis* Ames.

^b^ BNP, Białowieża National Park; BB, Biebrza National Park; JAS, the Jasienowka farm; DSMZ, German Collection of Microorganisms and Cell Cultures; WSBC, Weihenstephan *Bacillus* collection; BGSC, *Bacillus* Genetic Stock Center; ATCC, American Type Culture Collection.

^c^ +, the growth at 7 ^o^C was observed. The negative results were omitted.

^d^ +, *cry1*; +^2^, *cry2*; +^4^, *cry4.* The negative results were omitted.

^e^ +, the presence of the *cytK* gene tested in PCR. In parenthesis the relative expression calculated according to the method of Pfaffl [Pfaffl 2001]. The negative results were omitted.

^f^ ST, sequencing type. New STs are indicated with a N index.

^g^ The clades designation corresponds with Figure 5, and Figure 6.

^h^ CC, clonal complexes; S, singleton. The CCs designation corresponds with Figure 3, Figure 4, Figure S1, and Figure S2.
