# Supplementary figures and images for: Eco-Genetic Structure of Bacillus cereus sensu lato Populations from Different Environments in Northeastern Poland
Source: PLoS One. 2013 Dec 2;8(12):e80175. doi: 10.1371/journal.pone.0080175 (PMC3846478; doi:10.1371/journal.pone.0080175)

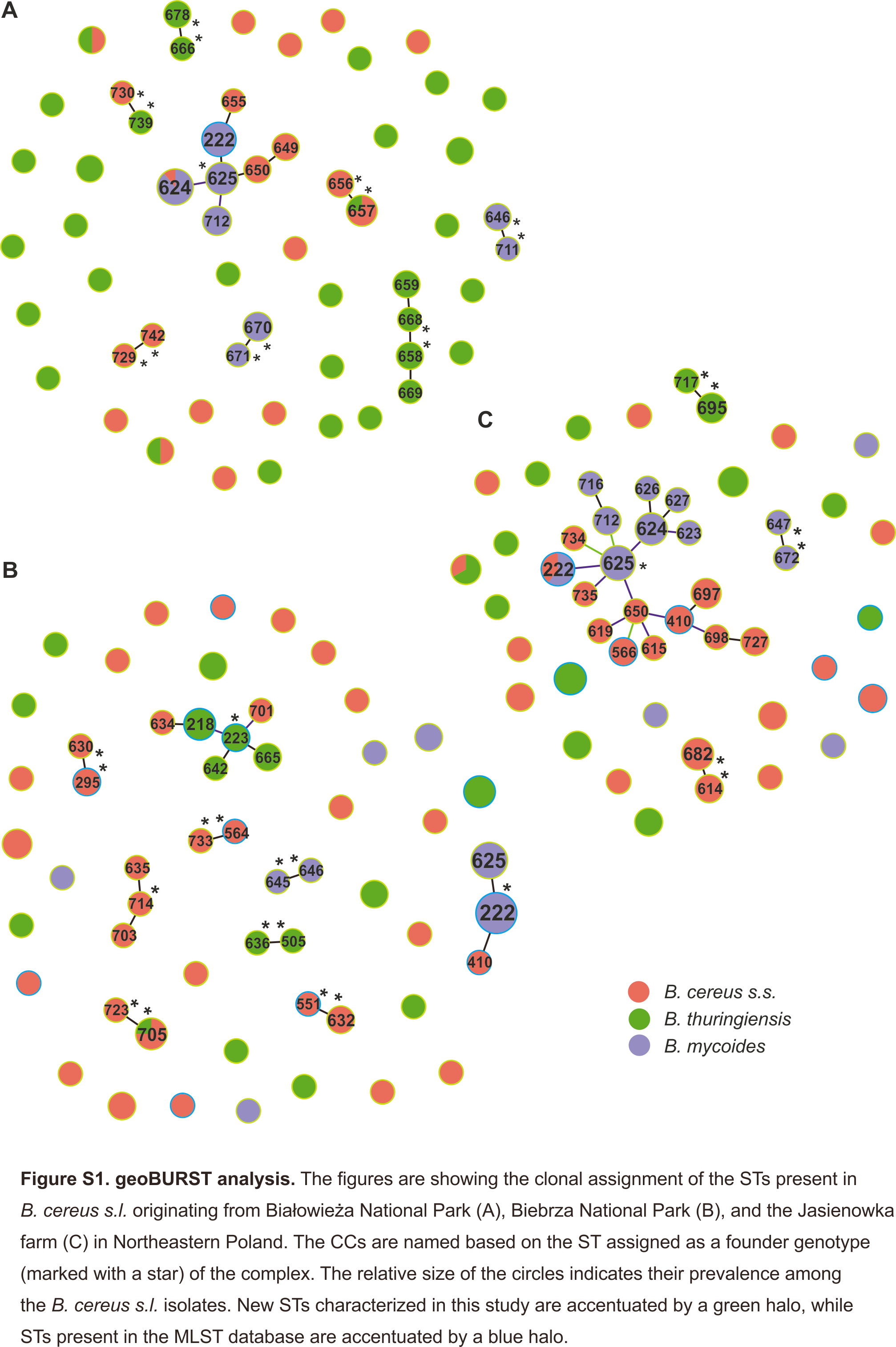

Supplement: Figure S1 — geoBURST analysis. The figures are showing the clonal assignment of the STs present in B. cereus s.l. originating from Białowieża National Park (A), Biebrza National Park (B), and the Jasienowka farm (C) in northeastern Poland. The CCs are named based on the ST assigned as a founder genotype (marked with a star) of the complex. The relative size of the circles indicates their prevalence among the B. cereus s.l. isolates. New STs characterized in this study are accentuated by a green halo, while STs present in the MLST database are accentuated by a blue halo. (TIF) [file pone.0080175.s005.tif]

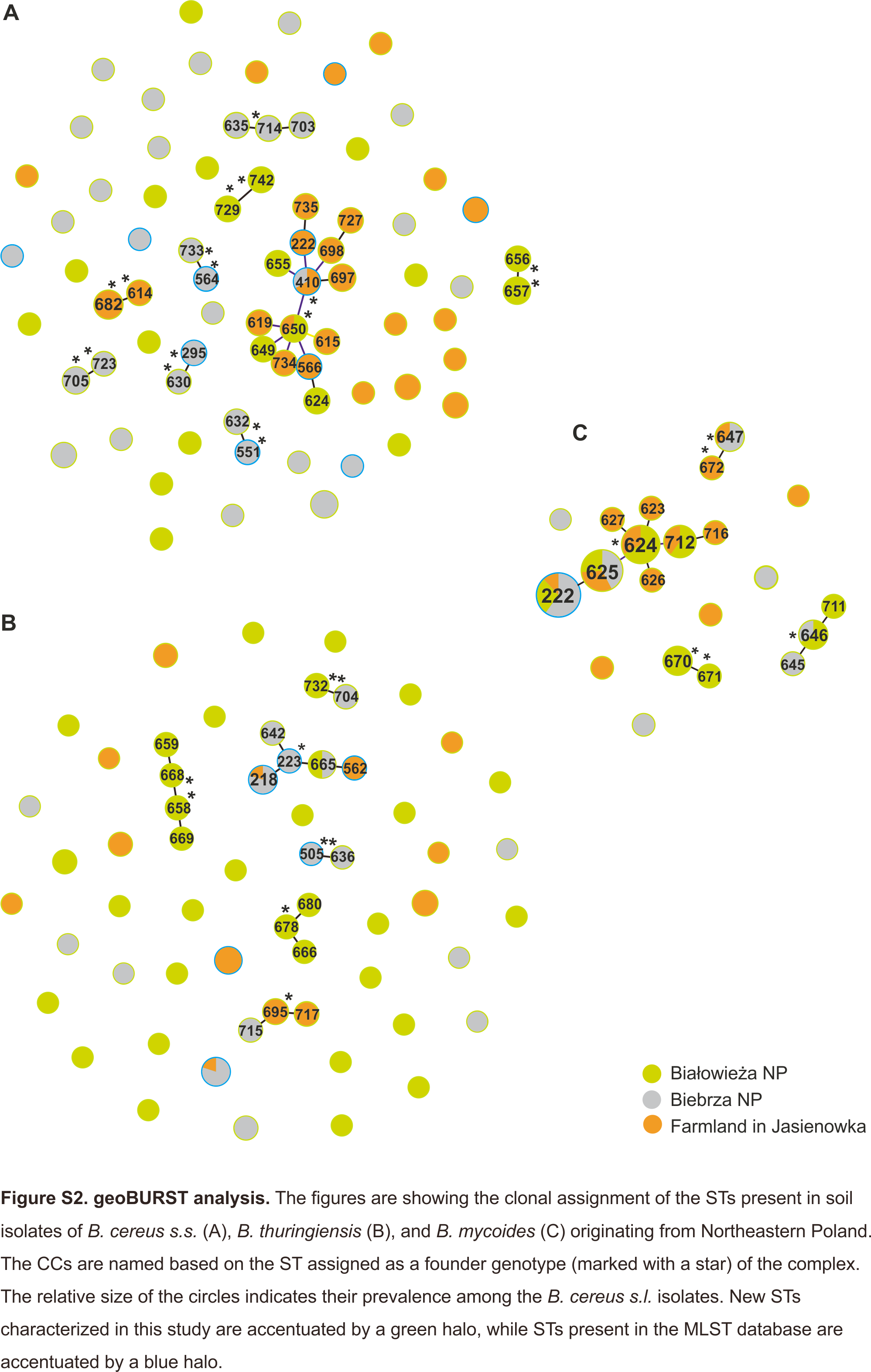

Supplement: Figure S2 — geoBURST analysis. The figures are showing the clonal assignment of the STs present in soil isolates of B. cereus s.s. (A), B. thuringiensis (B), and B. mycoides (C) originating from northeastern Poland. The CCs are named based on the ST assigned as a founder genotype (marked with a star) of the complex. The relative size of the circles indicates their prevalence among the B. cereus s.l. isolates. New STs characterized in this study are accentuated by a green halo, while STs present in the MLST database are accentuated by a blue halo. (TIF) [file pone.0080175.s006.tif]
